# COVID-19 Pandemic and Overall Mental Health of Healthcare Professionals Globally: A Meta-Review of Systematic Reviews

**DOI:** 10.3389/fpsyt.2021.804525

**Published:** 2022-01-17

**Authors:** Muhammad Chutiyami, Allen M. Y. Cheong, Dauda Salihu, Umar Muhammad Bello, Dorothy Ndwiga, Reshin Maharaj, Kogi Naidoo, Mustapha Adam Kolo, Philomina Jacob, Navjot Chhina, Tan Kan Ku, Liza Devar, Pratitha Pratitha, Priya Kannan

**Affiliations:** ^1^School of Nursing, Institute of Health and Management, Sydney, NSW, Australia; ^2^School of Optometry, The Hong Kong Polytechnic University, Kowloon, Hong Kong SAR, China; ^3^Centre for Eye and Vision Research, Hong Kong, Hong Kong SAR, China; ^4^School of Nursing, The Hong Kong Polytechnic University, Kowloon, Hong Kong SAR, China; ^5^Department of Physiotherapy, Yobe State University Teaching Hospital (YSUTH), Damaturu, Nigeria; ^6^Institute of Health and Management, Melbourne, VIC, Australia; ^7^Department of Geography, University of Maiduguri, Maiduguri, Nigeria; ^8^Health Careers International Pty Ltd., Kochi, India; ^9^Department of Rehabilitation Sciences, The Hong Kong Polytechnic University, Kowloon, Hong Kong SAR, China

**Keywords:** COVID-19, health professional, mental health, review–systematic, coping strategies

## Abstract

**Objective:**

This meta-review aimed to provide a comprehensive overview of overall mental health of healthcare professionals during the COVID-19 pandemic.

**Method:**

We conducted a comprehensive literature search on Academic Search Premier, CINAHL, Cochrane Library, and MEDLINE. A predefined eligibility criterion was used to screen the articles. The methodology quality of eligible studies was assessed using Joanna Briggs Institute checklist for systematic reviews. The data were narratively synthesised in line with the meta-review aim.

**Result:**

Forty systematic reviews (represented as *K* = 40), which reported data from 1,828 primary studies (*N*) and 3,245,768 participants, met the inclusion criteria. The findings from a pooled prevalence indicate that anxiety (16–41%, *K* = 30, *N* = 701), depression (14–37%, *K* = 28, *N* = 584), and stress/post-traumatic stress disorder (18.6–56.5%, *K* = 24, *N* = 327) were the most prevailing COVID-19 pandemic-related mental health conditions affecting healthcare workers. Other reported concerns included insomnia, burnout, fear, obsessive-compulsive disorder, somatization symptoms, phobia, substance abuse, and suicidal thoughts. Considering regions/countries, the highest anxiety was reported in the United-Kingdom [22.3, 95% Confidence Interval (CI):7–38, *N* = 4] compared to other countries, while the highest depression was in the Middle-East, (41, 95% CI:16–60, *N* = 5) and stress in the Eastern Mediterranean region (61.6, 95% CI:56.4–66.8, *N* = 2) compared to other regions. The most significant risk factors include female gender, younger age, being a nurse, and frontline professional. The most-reported coping strategies include individual/group psychological support, family/relative support, training/orientation, and the adequacy of personal protective equipment.

**Conclusion:**

It was concluded that healthcare professionals (nurses, doctors, allied health) have experienced various mental health issues during COVID-19 pandemic. The meta-review, therefore, recommends targeted interventions and health policies that address specific mental health issues to support health professionals worldwide during the duration of the COVID-19 pandemic and similar future health crises.

**Systematic Review Registration:**

https://www.crd.york.ac.uk/prospero/display_record.php?ID=CRD4202126200, identifier: CRD42021262001.

## Introduction

Coronavirus pandemic (COVID-19) has caused an unprecedented concern across the globe since the current outbreak began in 2019 in Wuhan, China (1). The outbreak was declared a pandemic by the World Health Organisation (WHO) in March 2020 (2). As of 4 September 2021, over 200 million cases and 4.5 million deaths have been reported across more than 200 countries/territories worldwide (2). The number of cases and mortalities continue to increase across different countries despite efforts to control and manage the threat. Recent mutations in the virus represent a constant concern, with new strains, such as the Bengal variant identified in India (3), leading to second and third waves of the disease transmission in multiple countries (2).

The COVID-19 pandemic has resulted in significant impacts not only among the general population and affected patients but also among the health professionals (interchangeably referred to as healthcare workers (HCWs) who care for infected patients. Although the pandemic has affected various aspects of health and well-being, mental health is among the most reported concerns (4–6). Countries that have experienced high caseloads, such as Italy (7) and Spain (8), have reported a higher prevalence of mental health issues among healthcare workers (HCWs) relative to less-affected regions. During the early stages of the outbreak, the highest prevalence of mental health concerns was reported in China, where the outbreak originated (4). Similar to the current COVID-19 outbreak, previous pandemics, including those associated with Severe Acute Respiratory Syndrome (SARS) and Middle East Respiratory Syndrome (MERS), were characterised as mental health disturbances in both the general population and among health professionals (9–11). The current COVID-19 pandemic has several aspects of psychiatric interest and relevance considering the uncertainties and hopelessness among the general population, of which efforts have not been successful in overcoming the outbreak (12). Marazziti and Stahl (12) added that psychiatrists could play a significant role in supporting nurses, doctors and other frontline professionals as well as managing the long-term consequences of the pandemic. Ghebreyesus (13) further necessitates the need for preparedness and getting services ready, particularly in resource-poor countries before another outbreak through supporting the countries in establishing community-based mental health services for everyone. Therefore, addressing the mental health needs of the general population at large and health professionals, in particular, is of paramount importance.

Many primary studies have been conducted to examine various mental health aspects among health professionals or the general population in different countries, including African (14), American (15), Asian (16–18), and the European (19–22) countries. Similarly, several systematic reviews have been conducted to summarise these mental health concerns among health professionals (23–26). Most systematic reviews have been conducted to explore specific aspects of mental health among health professionals, such as anxiety and depression (26–28), insomnia (29), and post-traumatic stress disorder (PTSD) (30, 31). Other systematic reviews have been conducted in specific categories of HCWs, such as nurses (32), dental professionals (33), or surgeons (10). Systematic reviews have also been limited to certain regions/countries, such as China (34). These systematic reviews have been conducted at different stages of the outbreak, focusing on different factors; the consolidation of these findings is of paramount importance to provide comprehensive evidence regarding the prevalence and risk factors associated with mental health issues among HCWs to guide policymakers and other stakeholders in the allocation of resources and interventions. This review attempted to summarise existing systematic reviews examining the impacts of the ongoing COVID-19 pandemic on various aspects of mental health among health professionals. The primary aim of the current systematic review of systematic reviews (termed a meta-review) was to provide a comprehensive overview of the overall mental health of healthcare professionals during the COVID-19 pandemic. Our secondary aim was to report coping strategies reported alongside the mental health problems to open windows for further studies. For the purposes of this article, the term COVID-19 is used interchangeably to refer to both COVID-19 and SARS-CoV-2 pandemic.

## Methods

A systematic review of systematic reviews (referred to as a meta-review) was adopted for this study. The reporting of this meta-review was guided by the standards established by the Preferred Reporting Items for Systematic Review and Meta-Analysis (PRISMA) extension statement (35). The review question was formulated using a PICO (Participants, Intervention, Comparator, Outcome) framework. The participants comprised HCWs, including nurses, medical doctors, and allied health professionals such as physiotherapists. For this review, the intervention was considered to be exposure to COVID-19, and the comparator group included members of the general population or non-health professionals. The assessed outcomes were the prevalence and risk factors of various mental health issues. The review was registered with the international prospective register of systematic reviews (PROSPERO: CRD42021262001).

### Eligibility Criteria

Studies were included if they were systematic reviews with or without meta-analyses; were published in the English language; could be obtained in full-text format; and assessed the impacts of COVID-19 among health professionals (medical doctors, nurses, allied health professionals). Scoping reviews and rapid reviews were included if they employed key systematic approaches to the review process, including a predefined search strategy, screening, data extraction, and synthesis. Systematic reviews that included the general population but performed a separate analysis of HCWs were included. Additionally, systematic reviews that synthesised data including previous pandemics but reported separate COVID-19-related findings were also included. Exclusion criteria included traditional literature reviews, narrative reviews (non-systematic), primary studies, non-COVID-19-related studies, and reviews assessing the COVID-19 impacts on non-health professionals.

### Information Sources

Four electronic databases, including Academic Search Database, CINAHL Complete, Cochrane Database of Systematic Reviews, and MEDLINE Complete, were searched for eligible studies examining the mental health impacts of COVID-19 pandemic among HCWs. The search was supplemented with a Google Scholar search (first 10 pages), and a “snowballing” approach was used to identify additional resources from reference lists and citations cheques. The search was not restricted by a publication start date, and all databases were searched until June 2021.

### Searches

A comprehensive search of each database was conducted using keywords/medical subheading (MeSH) terms to identify relevant systematic reviews. Boolean operators and truncations were also used. EBSCOHost was used to search Academic Search Database, CINAHL Complete, and MEDLINE Complete using the same search terms: (COVID-19 OR Coronavirus OR SARS-COV2) AND (“mental health” OR psychological OR depression OR post-trauma^*^ OR anxiety OR stress^*^ OR burnout OR insomnia OR suicide^*^) AND (“healthcare worker^*^” OR “medical staff” OR “health professional^*^” OR nurse^*^ OR physician^*^ OR “medical doctor”) AND (“systematic review” OR “rapid review” OR “scoping review”). Cochrane Database of Systematic Reviews was searched using the terms; (COVID-19 OR Coronavirus OR SARS-COV2) AND (“healthcare worker^*^” OR “medical staff” OR “health professional^*^” OR nurse^*^ OR physician^*^ OR “medical doctor”). The search of Google Scholar was conducted using the term “covid-19 healthcare worker mental health.” The search was limited to articles published in the English language.

### Selection of Evidence

The predefined eligibility criteria were applied to the selection process, which involved the sequential screening of the titles, abstracts, and full texts of the systematic reviews identified by the electronic database search. Three reviewers (MC, UMB, and PJ) screened and selected articles using the predefined inclusion and exclusion criteria. Two of the reviewers (MC and PJ) screened the studies independently and resolved discrepancies by discussion, while the third reviewer (UMB) was involved if an agreement was not reached. The selected studies were systematic reviews examining any aspect of mental health among health professionals during the COVID-19 pandemic.

### Data Extraction

Data extraction was performed using a Microsoft Excel package specifically designed to meet the aim of the review. The extraction form was designed by three reviewers (DS, UMB and MAK) and included author's details, the aims of the review/research question(s), types of primary studies included in the review, location of primary studies included in the review, type of health professionals (e.g., nurses) assessed in the review, specific mental health domains assessed, measures/instruments used for assessments, detailed results, and author's conclusions. Two reviewers (LD and PP) extracted the data from the included studies. Differences were resolved through discussion between the two authors. A third reviewer (MC) cross-checked all extracted data for accuracy and completeness.

### Critical Appraisal of the Included Studies

Quality appraisals of the included studies were performed using the Joanna Briggs Institute (JBI) checklist for systematic reviews (36). The instrument consists of 11 items that assess different aspects of a systematic review, each of which can be answered using the options “Yes,” “No,” “Unclear,” or “Not Applicable” (36). An appraisal of each included systematic review was conducted independently by two reviewers (PJ and NC). The outcomes of the two reviewers were cross-checked by a third reviewer (MC), and all discrepancies were resolved by the third reviewer through re-examining the article. For this review, the number of items receiving a “yes” answer for each study was counted and used to determine the quality of the review. Although the JBI checklist for systematic reviews does not provide a classification guideline for determining the study quality, we considered studies that satisfied at least 70% of the criteria (8 out of 11 items) to be of good quality.

### Synthesis of Results

A meta-analysis was deemed inappropriate for this meta-review, as some of the included studies were already meta-analysed. Conducting a meta-analysis on a review that includes a meta-analysis risks inflating the statistical significance of the results (37). Therefore, an in-depth narrative synthesis was conducted by four of the reviewers (MC, AMYC, DS, UMB).

The narrative synthesis involved a detailed examination of the narrative and numeric summary findings and the reported conclusions regarding the impacts of the COVID-19 pandemic on any aspect of mental health among health professionals, including the prevalence of mental health issues and associated risk factors among medical doctors, nurses, and allied health professionals. The impact of COVID-19 on the overall prevalence of mental health issues was reported for those studies that did not include a comparison with non-health professionals. For studies that reported a comparison against a non-healthcare population, the impact was reported as either significant or non-significant. Where available and possible, the effect sizes, study designs included in the systematic reviews (narrative synthesis or meta-analysis), and the quality of the systematic review was considered when drawing conclusions.

## Results

### Selection of Included Studies

**Figure 1:**
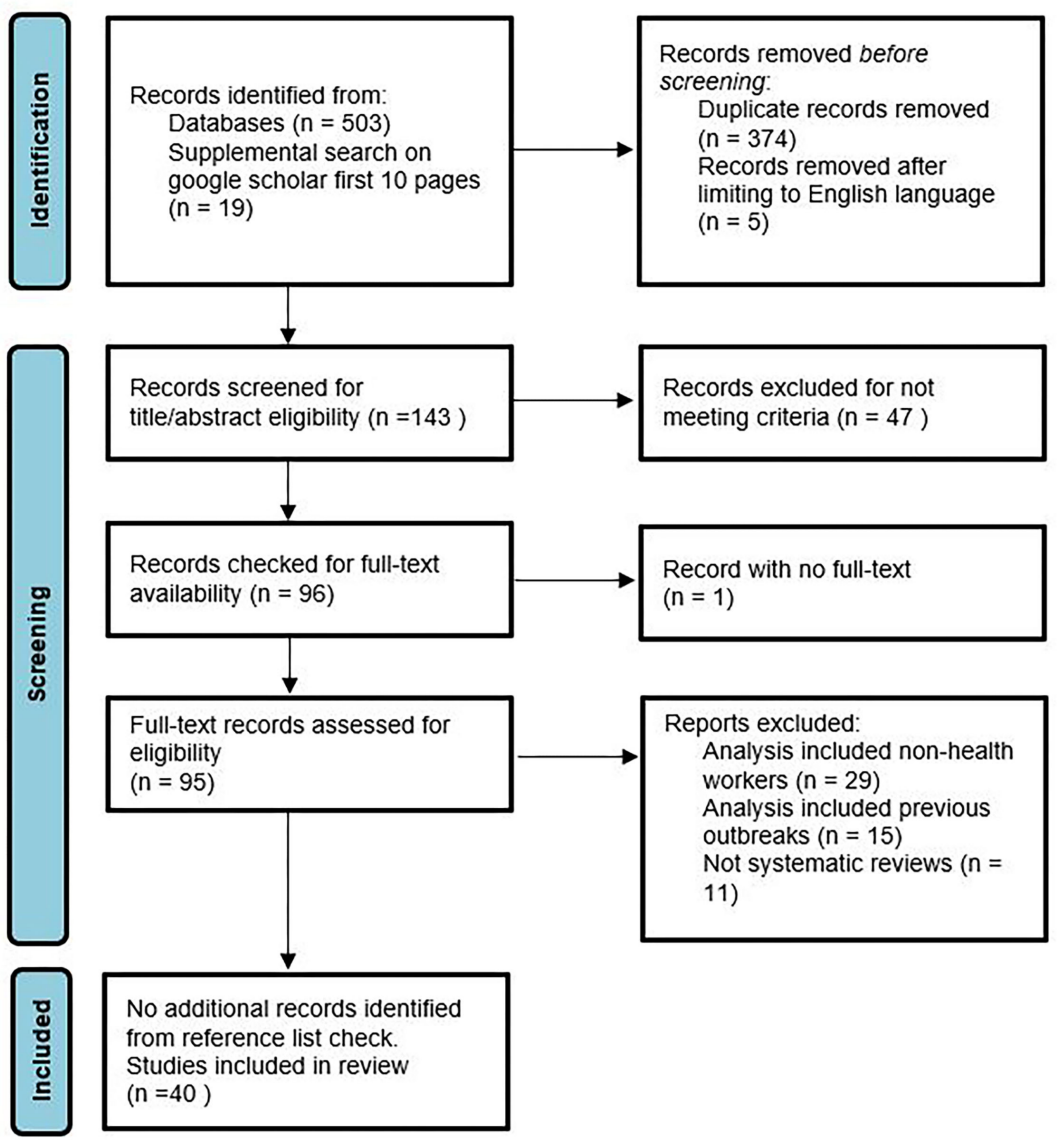
PRISMA flow chart indicating study selection process.

The study selection steps are reported in Figure 1. The initial search from the four databases (Academic Search Premier, CINAHL, MEDLINE, and Cochrane) resulted in the identification of 503 articles, and the supplemental search performed on Google Scholar resulted in 19 relevant articles, resulting in a total of 522 articles. Duplicate articles were removed, and an English language limitation was applied to the database search, which resulted in the identification of 143 articles. These 143 articles were screened according to titles and abstracts against the eligibility criteria, resulting in the identification of 96 articles that potentially met the inclusion criteria. One study without available full text was removed, and the full texts of the remaining 95 studies were retrieved and screened for eligibility. Finally, 40 studies were identified as fully meeting the eligibility criteria. The reference lists of these 40 studies were reviewed, which did not result in the identification of any additional studies. Therefore, 40 studies were included in the final review.

### Characteristics of the Included Studies

The 40 systematic reviews (represented as K) included in this meta-review were published between 2020 and 2021 (Supplementary Table 1). The total number of primary studies (represented as N) included in the systematic reviews was 1,828; however, three reviews (*K* = 3, 7.5%) included studies beyond COVID-19, such as those examining the impacts of SARS or MERS. A total of 3,245,768 subjects (represented as n) were included, although the majority of the systematic reviews did not report either genders or ages (*K* = 22, 55%); eight reported one but not the other (*K* = 8, 20%), and only ten reviews reported both (*K* =10, 25%). Eleven studies reported genders, with women (*n* = 468,851, 53.8%) constituting high proportion. Twelve studies reported an age range between 18 and 75 years. Ten studies reported on a mixture of health professionals and the general population (*n* = 2,204,914, 67.9%), whereas 30 studies included only health professionals with (*n* = 1,040,854, 32.1%). The most commonly used search databases among the included systematic reviews were PubMed (*K* = 29, 72.5%), MEDLINE (*K* = 20, 50%), Embase (*K* = 20, 50%), Web of Science (*K* = 14, 35%), PsycINFO (*K* = 12, 30%), Google Scholar (*K* = 10, 25%), Scopus (*K* = 10, 25%), and CINAHL (*K* = 8, 20%). The most commonly reported study design was cross-sectional (*K* = 32, 80%). The General Anxiety Disorder 7 (GAD-7, *K* = 28, 70%), Patient Health Questionnaire (PHQ, *K* = 26, 65%), Depression Anxiety Stress Scale (DASS, *K* = 21, 52.5%), Zung Self-Rating Depression Scale (SDS, *K* = 17, 42.5%), Zung Self-Rating Anxiety Scale (SAS, *K* = 17, 42.5%), Insomnia Severity Index (ISI, *K* = 16, 40%), Pittsburgh Sleep Quality Index (PSQI, *K* = 16, 40%) were the most commonly reported instruments used for the assessment of mental health and associated factors.

### Critical Appraisal of the Included Studies

The included systematic reviews were evaluated using quality assessment criteria, with scores ranging from 3/11 to 11/11 based on the JBI checklist (Table 1). The majority of the studies (31/40) were considered of good quality, which we defined as meeting at least 70% of the (8/11) assessment criteria. All included studies satisfied the first criterion of stating a clear and explicit research question or aim, whereas half (20/40) of the studies failed to meet the criterion of assessing publication bias. All studies were included in the synthesis of findings, regardless of their quality assessment score.

**Table 1:** Outcome of the critical appraisal of the included studies.

**S/ no**	**Study references**	**Criteria assessed based on JBI checklist**											**Total criteria met**
		**1**	**2**	**3**	**4**	**5**	**6**	**7**	**8**	**9**	**10**	**11**	
1	Al Maqbali et al. (32)	1	1	1	1	1	-	-	1	1	1	1	9
2	Arora et al. (23)	1	1	1	1	1	1	1	1	1	1	0	10
3	Cenat et al. (27)	1	1	1	1	1	1	1	1	1	1	1	11
4	De Brier et al. (38)	1	1	1	1	1	1	1	1	0	1	1	10
5	da silva Neto et al. (39)	1	1	1	1	1	1	1	1	1	0	0	9
6	da silva and Neto (40)	1	1	1	1	-	-	1	1	1	-	1	8
7	da silva and Neto (41)	1	1	1	1	1	1	-	-	-	1	1	8
8	Danet (42)	1	1	1	1	1	-	-	1	-	1	1	8
9	De Kock et al. (24)	1	1	1	1	1	1	1	1	-	1	1	10
10	De Pablo et al. (43)	1	1	1	1	1	-	1	1	0	1	0	8
11	D'Ettorre et al. (30)	1	1	1	0	1	-	1	1	0	1	0	7
12	Dong et al. (34)	1	1	1	1	1	1	1	1	1	1	1	11
13	Falasi et al. (31)	1	1	-	1	1	-	1	1	0	1	1	8
14	Galanis et al. (44)	1	1	1	1	1	0	0	1	1	1	0	8
15	Gohil et al. (33)	1	1	1	1	-	-	1	1	0	1	0	7
16	Hao et al. (45)	1	1	1	1	1	-	1	1	1	-	1	9
17	Krishnamoorthy et al. (46)	1	1	1	1	1	1	-	1	1	1	0	9
18	Kunz et al. (25)	1	1	-	0	1	1	0	1	0	1	0	6
19	Kunzler et al. (47)	1	1	1	1	1	1	1	1	-	1	1	10
20	Li et al. (48)	1	1	1	1	1	0	1	1	1	1	1	10
21	Luo et al. (49)	1	1	1	1	1	-	-	1	0	1	1	8
22	Mahmud et al. (28)	1	1	1	1	1	1	1	1	1	1	1	11
23	Marvaldi et al. (26)	1	1	1	1	1	-	1	1	1	1	1	10
24	Moitra et al. (50)	1	1	1	1	-	-	1	1	0	1	-	7
25	Muller et al. (51)	1	1	1	1	1	1	1	1	0	1	1	10
26	Pappa et al. (29)	1	1	1	1	1	1	1	1	0	1	1	10
27	Phiri et al. (52)	1	1	1	1	1	1	1	1	1	1	-	10
28	Salari et al. (53)	1	1	-	1	1	0	0	1	1	1	0	7
29	Sanghera et al. (54)	1	1	1	1	-	-	-	1	-	1	1	7
30	Santabarbara et al. (55)	1	1	1	1	1	1	-	1	1	1	-	9
31	Saragih et al. (56)	1	1	1	1	1	1	1	1	1	1	1	11
32	Sharifi et al. (57)	1	1	1	1	1	1	-	1	-	1	1	9
33	Shaukat et al. (58)	1	1	1	0	-	-	-	1	0	1	0	5
34	Sheraton et al. (59)	1	1	-	1	1	1	1	1	1	1	1	10
35	Sriharan et al. (60)	1	1	1	1	1	1	1	1	0	1	1	10
36	Thatrimontrichai et al. (61)	1	-	1	0	0	-	1	-	0	0	1	4
37	Varghese et al. (62)	1	1	1	1	1	1	1	1	1	1	1	11
38	Vindegaard and Benros (63)	1	1	0	0	-	0	0	-	0	0	1	3
39	Wu et al. (11)	1	1	1	1	1	-	1	1	1	1	-	9
40	Zhao et al. (64)	1	1	1	1	1	1	1	1	1	1	0	10

*Criteria 1 to 11- 1, clarity of review question; 2, appropriateness of inclusion criteria; 3, appropriateness of search strategy; 4, adequacy of search sources; 5, appropriateness for criteria in appraising included studies; 6, appraisal conducted by 2 or more reviewers independently; 7, methods to minimise errors in data extraction; 8, appropriate methods to combine studies; 9, assessment of publication bias; 10, recommendation for policy/practise based on reported data; 11, appropriateness of directives for new research. Key, 1, meet criteria; 0, Not meet criteria;, -, Unclear*.

### Study Findings

#### Overall Mental Health

Seven reviews, which synthesised data from 51 primary studies (*N* = 51), reported the overall mental health impacts of COVID-19 on HCWs (Table 2). Of these, the prevalence rate was assessed in four reviews, two of which reported pooled prevalence values calculated from meta-analyses, ranging from 11.6% [95% confidence interval (CI): 9.2–14.6%, *N* = 3] (64) to 34% (95% CI: 24–44%, *N* = 28) (23). One review (40) reported a positive correlation between COVID-19 and the incidence of psychiatric disorders (*N* = 8).

**Table 2:** Mental health impacts of COVID-19 on health professionals.

**Outcomes**	**Measure**	**References**	**Impact of COVID-19 on outcome**			**Effect size/comment**
			**Impact classified/**		**Overall impact**	
			**compared between groups**		**(no comparison)**	
			**Significant**	**Not significant**		
Overall mental health/ psychological problems	BAI, CES-D, CPDI, DASS-21, GAD-7, GHQ-12; HADS-A, HAMA, HAMD, IES-R, ISI, ITQ, PHQ-9: PTSD-SS, PSQI, SAS, SASR, SDS, SOS, SRQ, STAI, WHO-5	Arora et al. (23)			✓	34% (95%CI: 24–44) *N* = 28
	NA	De Brier et al. (38)	✓			β: 5.347, (95%CI:3.831;8.184) *N* = 1. Contact with infected patients
	GAD-7, GHQ, PHQ-4, PHQ-9, SCL-90,	da Silva and Neto (40)	✓			Meta-correlation between covid and psychiatric disorder = 0.72% (95%CI: 0.66–0.78) *N* = 8
	NA	Luo et al. (49)			✓	Range = 14 to 72%, *N* = 5
	NA	Shaukat et al. (58)			✓	23% *N* = 1
	NA	Sheraton et al. (59)		✓		OR = 1.39 (95%CI: 0.99–1.96), Z = 1.89 *N* = 5. compared to non-HCW
	NA	Zhao et al. (64)			✓	11.6% (95% CI: 9.2–14.6) *N* = 3, *n* = 3,327
Anxiety/ Anxiety symptoms	#GAD-7, SAS	Al Maqbali et al. (32)			✓	37% (95% CI 32–41), *N* = 73. Nurses only
	NA	De Brier et al. (38)	✓			AOR: range from 1.57 to 2.06, *N* = 2 Contact with infected patients
	BAI, DASS-21, GAD-7, GAD-2, HAMA, SAS,	Cenat et al. (27)		✓		16% (95%CI:12–20) *N* = 23, > 15% (95%CI:11–20) *N* = 31
	AS, DAS, GAD-7, HAMA, SAS, SCL-90, SF-36	da Silva Neto et al. (39)	✓			13%, OR = 1.62 (95%CI:1.33–1.96) *N* = 7, higher than non-HCW, 5%
	DASS-21, GAD-7, SF-36, STAI	Danet (42)			✓	Range = 20–72%, *N* = 7
	DASS-21, GAD-7	De Kock et al. (24)			✓	Range = 14.5–44.6%, *N* = 2
	NA	de Pablo et al. (43)	✓			22.2% (95%CI: 13–36) *N* = 4, *n* = 7,716
	DASS-21, GAD-7, SAS	Dong et al. (34)			✓	34.4% (95%CI: 30–39) N = 22. China
	DASS-21, GAD-7, HAMA, SAS, SLC-90	Hao et al. (45)			✓	28.6% (95%CI: 22–36) *N* = 16
	NA	Krishnamoorthy et al. (46)			✓	24% (95%CI: 16–32) *N* = 16
	NA	Kunz et al. (25)			✓	65.2% N = 1. Only highest prevalence reported (Italy)
	NA	Kunzler et al. (47)		✓		SMD = −0.08 (95%CI: −0.66–0.49) *N* = 13, *n* = 5,508. compared to before covid
	NA	Luo et al. (49)			✓	26% (95%CI: 18–34) *N* = 12
	# BAI, DASS-21, HAMA, HADS, GAD, SAS	Mahmud et al. (20)			✓	41.42% (95% CI: 36–47) *N* = 75, *n* = 147,435
	NA	Marvaldi et al. (26)			✓	30% (95 %CI, 24.2–37.05) *N* = 22, *n* = 51,942
	NA	Moitra et al. (50)			✓	Not quantified. *N* = 10
	NA	Muller et al. (51)			✓	24% (95%CI: 9–90) *N* = 22, *n* = 47,630
	BAI, DASS-21, HAMA, GAD-7, SAS	Pappa et al. (29)			✓	23.2% (95%CI: 18–29) *N* = 12
	DASS-21, GAD-7, HADS	Phiri et al. (52)			✓	21.9% (95%CI: 19-25) N= 69
	DASS-21, GAD-7, SARS, SAS	Salari et al. (53)			✓	25.8% (95% CI 20.5–31.9%) *N* = 23
	DASS-21, GAD-7, HAMA, SAS	Sanghera et al. (54)			✓	Range = 12.3–35.6% *N* = 33
	BAI, DASS-21, GAD-7, HADS, STAI-S, SAS	Santabarbara et al. (55)			✓	25% (95% CI: 21–29%) *N* = 71
	NA	Saragih et al. (56)			✓	40% (95% CI: 29–52%) *N* = 40
	DASS-21, GAD-2/7, HADS, HAMA, PHQ-4, SAS	Li et al. (48)			✓	22.1% (95% CI, 18.2–26.3%) *N* = 57
	GAD-7, SAS	Shaukat et al. (58)			✓	Range = 23–44% *N* = 2
	NA	Thatrimontrichai et al. (61)			✓	25.9%, *N* = 18, *n* = 6,305/24,297. Asia
	NA	Varghese et al. (62)			✓	32% (95%CI: 21–44%) *N* = 21, *n* = 13 641. Nurses
	NA	Vindegaard and Benros (63)			✓	Not quantified. *N* = 8.
	NA	Wu et al. (65)			✓	29% (95%CI 23.6–34.7) *N* = 23, *n* = 50,143 Nurses/doctors; 19.9% (12.4–28.6) *N* = 7, n = 2,521 other professionals
	NA	Zhao et al. (64)			✓	23.2% (95% CI: 17–31) *N* = 14, *n* = 13,020
Burnout	MBI	Danet (42)			✓	Range = 12–36% (emotional exhaustion and depersonalisation) *N* = 2
	NA	de Pablo et al. (43)	✓			25% (95%CI: 13–43) *N* = 1, *n* = 32
	NA	Galanis et al. (44)			✓	emotional exhaustion 34.1%, depersonalisation 12.6%, lack of personal accomplishment 15.2%; *N* = 6. Nurses
	NA	Kunz et al. (25)			✓	45.6%, *N* = 1. Only highest prevalence reported (Belgium)
	NA	Moitra et al. (50)			✓	Not quantified. *N* = 2
	MBI	Sanghera et al. (54)			✓	Range = 3.1–43.0%, *N* = 5
	MBI, questionnaire, Pfi	Sharifi et al. (57)			✓	Not quantified. *N* = 12
	MBI, questionnaire	Sriharan et al. (60)			✓	Range = 13–39%, *N* = 2. Nurses
Depression/ depressive symptoms	#PHQ-9, SDS	Al Maqbali et al. (32)			✓	35% (95%CI: 31–39) *N* = 62, nurses
	NA	De Brier et al. (38)	✓			AOR: range from 1.52 to 2.97, *N* = 2. Contact with infected patients.
	BDI, DASS-21, HAMD, PHQ-2, PHQ-9, SDS	Cenat et al. (27)		✓		14% (95%CI:11–17) *N* = 18, < general population 17% (95%CI:13–22) *N* = 28
	DS, HAMD, PHQ-4, PHQ-9, SDS	da Silva Neto et al. (39)	✓			12.2%, OR = 1.3246; 95%CI 1.0930 to 1.6053) *N* = 7, > other professionals 9.5%
	DASS-21, IPQ, PHQ-9, SDS	Danet (42)			✓	Range = 25–65%, *N* = 10
	DASS-21, PHQ-9	De Kock et al. (24)			✓	Range = 8.9–50.4% *N* = 2
	Estimate	de Pablo et al. (43)	✓			17.9% (95%CI: 7–40) *N* = 4, *n* = 7,716
	DASS-21, PHQ-9, SDS	Dong et al. (34)			✓	31.1% (95 CI: 25–38) *N* = 18. China
	DASS-21, HAMD, PHQ-2, PHQ-9, SCL-90, SDS	Hao et al. (45)			✓	24.1% (95% CI: 16–32) *N* = 14
	NA	Krishnamoorthy et al. (46)			✓	25% (95%CI:19–32) *N* = 16
	NA	Kunz et al. (25)			✓	57.9%, *N* = 1. Only highest prevalence reported (Italy)
	NA	Kunzler et al. (47)		✓		SMD =-0.16 (95%CI:−0.59–0.26) *N* = 7, *n* = 2,226. compared to before covid
	#SDS, CES-D, DASS-21, HADS	Mahmud et al. (20)			✓	37.12% (95% CI:32–42) *N* = 69, *n* = 144,649
	NA	Marvaldi et al. (26)			✓	31% (95 %CI, 26–37) *N* = 25, *n* = 68,030
	NA	Moitra et al. (50)			✓	Not quantified. *N* = 18
	NA	Muller et al. (51)			✓	28% (95%CI: 5–51) *N* = 19, *n* = 35,219
	BDI-II, DASS-21, CES-D, PHQ-2, SDS	Pappa et al. (29)			✓	22.8% (95%CI: 15–32) *N* = 10
	DASS-21, HADS, PHQ-9	Phiri et al. (52)			✓	23.4% (95%CI: 21–26) *N* = 66
	DASS-21, SDS, BDI-II, HAD	Salari et al. (53)			✓	24.3% (95%CI: 18–32%) *N* = 21
	DASS-21, PHQ-9, PHQ-4, SDS, HAMD	Sanghera et al. (54)			✓	Range = 13.5–44.7%, *N* = 32
	NA	Saragih et al. (56)			✓	37% (95% CI: 29–45%) *N* = 30
	CES-D, DASS-21, HADS, PHQ-2, PHQ-4, PHQ-9	Li et al. (48)			✓	21.7% (95% CI:18–25) *N* = 55
	NA	Shaukat et al. (58)			✓	50.4%, *N* = 1
	NA	Thatrimontrichai et al. (61)			✓	27.2%, *N* = 14, *n* = 10,617/39,014. Asia
	NA	Varghese et al. (62)			✓	32% (95% CI: 21–44) *N* = 17, *n* = 12 294
	NA	Vindegaard and Benros (63)			✓	Not quantified. *N* = 6
	#GHQ-9, SDS, WHO-5	Wu et al. (65)			✓	31% (95%CI:25–38) *N* = 23, *n* = 41,889 Nurses/doctors; 14.1% (7.4–22.4) *N* = 6, *n* = 2,471 other professionals
	NA	Zhao et al. (64)			✓	23.9% (95% CI: 15–36) *N* = 11, *n* = 11,922
Fear	NA	De Brier et al. (38)	✓			AOR: 1.41, (95%CI:1.03;1.93), *N* = 1. Contact with infected patients.
	Self-questionnaire	De Kock et al. (24)			✓	87%, *N* = 1. Dentist. Fear of infection from patient or co-worker
	NA	Gohil et al. (33)			✓	Range = 60–96.6%, *N* = 12; Dental. Fear of contagion
	NA	Thatrimontrichai et al. (61)			✓	77.1%, *N* = 4, *n* = 2,743/3,558. Asia
Insomnia	AIS, ISI, PSQI	Cenat et al. (27)	✓			37% (95%CI:33–40) *N* = 6, HCW, higher than general population 16% (95%CI:8–30) *N* = 8
	ISI	da Silva Neto et al. (39)			✓	Range = 34–38.4%, *N* = 3
	ISI	De Kock et al. (24)			✓	34%, *N* = 1.
	NA	de Pablo et al. (43)			✓	44.5% (95%CI: 38–51) *N* = 3, *n* = 3,490
	ISI-7, PSQI	Hao et al. (45)			✓	44.1% (95% CI:31.3–57.0%) *N* = 5
	NA	Krishnamoorthy et al. (46)			✓	37% (95%CI:32–42) *N* = 4
	AIS, ISI, PSQI	Mahmud et al. (20)			✓	43.76% (95% CI: 36–52) *N* = 21, *n* = 33,370
	NA	Moitra et al. (50)			✓	Not quantified. *N* = 10
	AIS, ISI	Pappa et al. (29)			✓	38.9% (95%CI: 27–42) *N* = 5
	NA	Phiri et al. (52)			✓	23.98% (95%CI: 16–32) *N* = 4
	AIS, ISI, PSQI	Sanghera et al. (54)			✓	Range = 33.8–36.1%, *N* = 12
	ISS, PSQI	Shaukat et al. (58)			✓	34%, *N* = 1
	NA	Sheraton et al. (59)	✓			OR = 2.19 (95%CI: 1.33–3.62), Z = 3.08 *N* = 2. compared to non-HCW
	NA	Thatrimontrichai et al. (61)			✓	35%, *N* = 3, *n* = 2,072/5,919. Asia
	NA	Varghese et al. (62)			✓	38.3%, (95% CI = 5.8%−78.6) *N* = 2, *n* = 261
	NA	Wu et al. (65)			✓	47.3% (95%CI:39–56) *N* = 7, *n* = 13,375 Nurses/doctors; 31.8 (27.2–36.5) *N* = 2, *n* = 1,380 other professionals
Obsessive compulsive symptoms	NA	Hao et al. (45)			✓	16.2% (95%CI: 3.0–30) *N* = 4
	NA	Vindegaard and Benros (63)			✓	Not quantified. *N* = 1
Phobia	SLC-90, SCL	Hao et al. (45)			✓	35.0% (95% CI: 8.6–61) *N* = 4
PTSD/ emotional stress/ distress	NA	De Brier et al. (38)	✓			AOR: 1.60, (95%CI:1.25;2.04), *N* = 1. PTSD. Contact with infected patients.
	IES-R, K-6, SCL-90, SRQ-20	Cenat et al. (27)		✓		21% (95%CI:5–57) *N* = 4, HCW PTSD < general population 22% (95%CI:8–50) *N* = 9; 17% (95%CI:13–22) *N* = 9, HCW distress > general population 10% (95%CI:5–21) *N* = 10
	ASDI, IES-R; PSS	Al Maqbali et al. (32)			✓	43% (95% CI: 37–49), *N* = 40, nurses. Emotional stress
	NA	da silva and Neto (41)			✓	Not quantified, *N* = 31. HCW stress in ICU
	DASS-21, DSM-5, ASAISTSS	Danet (42)			✓	Range = 37–78% *N* = 10. stress
	NA	de Pablo et al. (43)			✓	29.9% (95%CI: 9–65) *N* = 3, *n* = 6,789. Distress; 7.7% (95%CI: 6–11) *N* = 22, *n* = 470 PTSD
	DASS-21, IES-R, IES-6, PCL-C, PTSD-SS	Dong et al. (34)			✓	29.1% (95%CI: 24–34) *N* = 9. Stress & PTSD, China
	CBI, GPS, IES-R, PCL-6, PCL-C	d'Ettorre et al. (22)			✓	Range = 6.6%-58.6%. *N* = 16, PTSD
	NA	Falasi et al. (31)			✓	Range = 3.4% (India) to 71.5% (China) *N* = 5. Acute PTSD
	IES-R, PTSD-SS, PCL-C, PSS-10	Hao et al. (45)			✓	25.6% (95% CI: 12–39) *N* = 5. PTSS
	NA	Krishnamoorthy et al. (46)			✓	41% (95% CI:19–65) *N* = 4 distress; 13% (11–16%) *N* = 2. PTSS
	NA	Kunz et al. (25)			✓	73.6% *N* = 1. Only highest prevalence reported (Spain). PTSD
	NA	Kunzler et al. (47)		✓		SMD = 0.49 (95% CI:−0.60–1.57) *N* = 3, *n* = 1,570. compared to before covid. Stress
	IES, DASS-21, PSS, PTSD	Mahmud et al. (20)			✓	44.86% (95% CI: 36.98–52.74) *N* = 41, *n* = 82,783. Stress
	NA	Marvaldi et al. (26)			✓	20.2% (95 %CI:9.9–33) *N* = 6 PTSD; 56.5% (95 %CI:31–81), *N* = 3 Acute stress
	NA	Muller et al. (51)			✓	37% (95%CI: 7–97) *N* = 13, *n* = 20,391
	IES-R, PCL-5	Phiri et al. (52)			✓	25% (95%CI: 19–31) *N* = 19. PTSD
	CES-D, IES-R, PSS-10, PSS	Sanghera et al. (54)			✓	Range = 5.2–32.9% *N* = 11 acute stress; 7.4–37.4% *N* = 13. PTSD
	NA	Saragih et al. (56)			✓	49% (95% CI: 22–75) *N* = 7 PTSD; 37% (95% CI: 25–50) *N* = 15 Distress
	NA	Li et al. (48)			✓	21.5% (95% CI, 1–35%) *N* = 9
	IES, PTSD-SS	Shaukat et al. (58)			✓	Range = 23.4–71%, *N* = 2. Stress disorder
	NA	Varghese et al. (62)			✓	18.6% PTSD (95% CI = 4.8%−38) *N* = 3, *n* = 638; 40.6% stress (95% CI = 25.4–56.8%,) *N* = 10, *n* = 4,204. Nurses
	#GHQ-12, IES, K6, PSS-10	Wu et al. (65)			✓	41.2 (19.8–64.5) *N* = 5, *n* = 10,165. Distress
	NA	Zhao et al. (64)			✓	28% (95% CI: 9.5–59) *N* = 5, *n* = 4,327. PTSS
Somatization symptoms	NA	Hao et al. (45)			✓	10.7% (95% CI: 1.9–19.6%) *N* = 5
	NA	Kunz et al. (25)			✓	Not quantified. *N* = 1. Reported as higher among nurses than doctors (Italy)
Substance abuse	NA	Kunz et al. (25)			✓	6.2% *N* = 1. Only highest prevalence reported in nurses and doctors (Spain)
Suicidal thought/ self-harm	NA	Phiri et al. (52)			✓	5.8% (95%CI: 5–7) *N* = 4

*# other measures not specified; N, number of studies; n, number of participants; AOR, Adjusted Odds Ratio; ASDI, Acute Stress Disorder Inventory; BAI, Becks Anxiety Inventory; BDI, Beck Depression Inventory; CES-D, Centre for Epidemiology Scale for Depression; CPDI, COVID-19 Peritraumatic Distress Index; DASS-21, Depression, Anxiety Stress Scale; DSM-5, PTSD Symptoms Severity Scale; GAD-7, Generalised Anxiety Disorder; GHQ, General Health Questionnaire; HADS-A, Hospital Anxiety Depression Scale–Anxiety; HAMA, Hamilton Anxiety Scale; HAMD, Hamilton Depression Scale; HCW, Healthcare Workers; IES-R, Impact of Event Scale-Revised; ISI, Insomnia Severity Scale; IPQ, Illness Perception Questionnaire; ITQ, International Trauma Questionnaire; K-6, Kessler-6 Item Psychological Distress Scale; MBI, Maslach Burnout Inventory; OR, Odds Ratio; PHQ, Patient Health Questionnaire; Pfi, Stanford Professional Fulfilment Index; PTSD-SS, Post Traumatic Stress Disorder-Short Scale; PTSS, Posttraumatic Stress Symptoms; PSQI, Pittsburgh Sleep Quality Index; PCL-C, PTSD Checklist Civilian; PTSD-SS, Posttraumatic Stress SAS, Self-rating Anxiety Scale; SASR, Stanford Acute Stress Reaction; SCL, Symptoms Checklist; SDS, Self-rating Depression Scale; SF, Health Questionnaire; SMD, Standardised Mean Difference; SOS, Stress Overload Scale; STAI, State-Trait Anxiety Inventory; SRQ, Stress Response Questionnaire; SRQ-20, Self Reporting Questionnaire-20; STSS, Secondary Traumatic Stress Scale; WHO-5, World Health Organization-5*.

Overall mental health risk factors include being a woman (58, 61) and being divorced (61). Compared with non-HCWs, health professionals reported a higher rate of mental health problems (23, 24, 39). Among health professionals, nurses (24, 58, 61) and doctors (40) were associated with the highest risk of developing any mental health problem. Additionally, longer working hours (61), fewer years of working experience (61), a lack of access to personal protective equipment (PPE) (61) and close contact with infected patients (41, 61) were associated with a higher incidence of mental health problems.

#### Anxiety

Anxiety or anxiety symptoms were assessed in 30 reviews, which synthesised data from 701 primary studies (Table 2). Of these, the prevalence rate was reported in 26 reviews, including 20 reviews that reported pooled prevalence values calculated from meta-analyses, ranging from 16% (95% CI: 12–20%, *N* = 23) (27) to 41.42% (95% CI: 36–47%, *N* = 75) (28). Among reviews without meta-analysis, the prevalence rate was estimated to be as high as 65.2% in Italy (25). The most-reported anxiety assessment tool was the GAD-7, which was reported in 15 reviews (Table 2).

The sociodemographic risk factors associated with the incidence of anxiety or anxiety-like symptoms included female gender (24, 29, 42, 45, 50, 58, 62), living in a rural area (24), being married (62), having a child (62), and younger age (≤40 years) (24, 39, 42, 50, 52, 62). Additionally, pre-existing illness (24), having physical COVID-19 symptoms (62), exposure to a COVID-19 patient (38, 48, 54, 62), working in a COVID-19 unit or hospital (62), working in an intensive care unit (ICU) (50), a lack of social support (54, 62), a lack of access to adequate PPE (54, 62), and insufficient knowledge regarding COVID-19 (54) were also associated with increased anxiety and anxiety-like symptoms.

The risk of developing anxiety was higher among nurses (29, 34, 42, 45, 50, 53, 55, 58, 65), and frontline professionals (24, 34, 42, 45, 50, 63, 65). The prevalence of anxiety among frontline nurses (39%, 95% CI: 32–46%, *N* = 24) was higher than among other nurses (32%, 95% CI: 27–38%, *N* = 42) (32) and overall health professionals (29.0%, 95% CI: 23.4–34.7%, *N* = 22) (34). Compared with the pre–COVID-19 prevalence, anxiety significantly increased during the COVID-19 pandemic (50). Health professionals with pre-existing insomnia were significantly more prone to developing anxiety symptoms [odds ratio (OR): 13.6, 95% CI: 10.5–17.5] (39).

Study location appears to contribute to the levels of anxiety reported among HCWs. In China, the prevalence of anxiety in Hubei Province, where the outbreak originated, was 37.9% (95% CI: 28.7–47.1%), which was higher than in other regions of China (30.8%, 95% CI: 25.1–36.5%) (34). Three reviews (48, 52, 62) conducted sensitivity analyses according to country or region. Phiri et al. (52) indicated that a higher incidence of anxiety was reported in the United Kingdom (UK: 22.3%, 95% CI: 7–38%, *N* = 4) compared with the United States of America (USA: 19.99%, 95% CI: 17%−23%, *N* = 4), China (18.98%, 95% CI: 16–22%, *N* = 24), and Italy (13.44%, 95% CI: 6–20%, *N* = 6). Li et al. (48) by contrast, reported that the Middle-East presented with the highest pooled estimated prevalence of anxiety (28.9%, 95% CI: 21.6–36.8%, *N* = 7), whereas, the lowest incidence was reported for North America (14.8%, 95% CI: 13.9–15.7%, *N* = 2). In Asia, China yielded a pooled prevalence of 19.1% (95% CI: 15.5–23.0%, *N* = 37), which was slightly lower than the pooled prevalence reported for all other studies from East Asia (20.5%, 95% CI: 15.7–25.8, *N* = 40). Other regions examined included Europe (23.9%, 95% CI: 19.6–28.4%, *N* = 4) and South Asia (21.0%, 95% CI: 11.7–31.4%, *N* = 3). Varghese et al. (62) examined the pooled prevalence among nurses across various regions and reported the highest pooled prevalence for the Eastern Mediterranean region (41.9%, 95% CI: 10.7–77.3%, *N* = 3, *n* = 907) compared with the Western Pacific/Southeast region (30.9%, 95% CI: 17.2–46.5%, *N* = 10, *n* = 10,579) and the European region (30.5%, 95% CI: 16.7–46.3%, *N* = 7, *n* = 2,067) (62).

#### Depression

Depression and depressive symptoms were assessed in 28 reviews, which synthesised data from 584 primary studies (Table 2). The prevalence rate was reported in 24 reviews, including 17 that reported the pooled prevalence values calculated from meta-analyses, which ranged from 14% (95% CI: 11–17%, *N* = 18) (27) to 37.12% (95% CI: 32–42%, *N* = 69) (28). Among reviews without meta-analyses, the prevalence rate was estimated to be as high as 65% (42). The most-reported depression assessment tools were the PHQ, versions 2 and 9, which were reported in 10 reviews (Table 2).

Exploring sociodemographic risk factors associated with depression revealed that female gender (24, 29, 42, 45, 50, 62), being single or not married (42), and younger age (≤40 years) (24, 39, 50, 52, 62) were associated with a higher incidence of depressive symptoms. Additionally, spending too much time reading COVID-19-related information (50), less work experience (42), a lack of social support (48), and pre-existing organic illnesses were associated with higher levels of depression (24). The risk of developing depression or depressive symptoms was higher among nurses (29, 42, 50, 65), frontline professionals (24, 42, 50, 63, 65), professionals working in surgical units (24), COVID-19 units and hospitals (62), and professionals with direct patient contact (38, 48, 54, 58). Depression was significantly associated with poor sleep quality and insomnia (39, 50). Health professionals with insomnia had a 13-fold higher risk of developing depressive symptoms than those without insomnia (OR: 13.5517, 95% CI: 10.4771–17.5285, *p* < 0.0001) (39).

Compared with the pre-COVID-19 prevalence, depressive symptoms significantly increased during the COVID-19 pandemic (50). The prevalence of depression among frontline nurses (33%, 95% CI: 24–43%, *N* = 19) was higher than that among other nurses (33%, 95% CI: 29–37%, *N* = 36) (32) and that among overall health professionals (29.2%, 95% CI: 21.7–36.7%) (34). Similarly, the prevalence of moderate to severe depression among frontline HCWs (14.6%, 95% CI: 6.3–23.0%) was higher than that among second-line HCWs (8.7%, 95% CI: 3.9–13.4%) (45).

Three reviews (48, 52, 62) conducted sensitivity analyses according to country or region. Phiri et al. (52) indicated that the highest depression prevalence was reported for the Middle East (41%, 95% CI: 16–60%, *N* = 5) compared with those reported for China (22.13%, 95% CI: 18%−27%, N = 24), Italy (20.39%, 95% CI: 10–31%, *N* = 5), and the UK (19.29%, 95% CI: 7%−32%, N = 5). Li et al. (48) also reported higher depression prevalence in the Middle East (34.6%, 95% CI: 25.1–44.9%, *N* = 5) compared with those in South Asia (28.8%, 95% CI: 18.1–40.8%, *N* = 3) and Europe (22.0%, 95% CI: 18.9–25.3%, *N* = 4). The pooled estimates were lowest for North America (18.7%, 95% CI: 17.8–9.7%, *N* = 2) and East Asia (19.1%, 95% CI: 15.2–23.4%, *N* = 39). Varghese et al. (62) examined the pooled prevalence of depression among nurses across various regions. The highest prevalence of depression was found in the Eastern Mediterranean region (61.2%, 95% CI: 16.9–96.2%, *N* = 2, *n* = 592) compared with the Western Pacific/Southeast region (27.4%, 95% CI: 13–44.7%, *N* = 9, *n* = 11,181) and European region (30.9%, 95% CI: 20.4–42.5%, *N* = 5, *n* = 433) (62).

#### PTSD/Stress/Distress

Emotional stress, distress, and PTSD were assessed from 24 reviews, which synthesised data from 327 primary studies (Table 2). Of these, the prevalence rate was reported by 21 reviews, including 15 that reported pooled prevalence values calculated from meta-analyses, ranging from 18.6% (95% CI: 4.8–38%, *N* = 3) (62) to 56.5% (95% CI: 31–81%, *N* = 3) (62). Among reviews without meta-analysis, the prevalence rate was estimated to be as high as 78% (42). The most-reported distress and PTSD assessment tool was the Impact of Event Scale (IES), which was reported in 10 reviews (Table 2).

The risk of developing PTSD, stress, or distress was generally higher among women (30, 31, 42, 50, 62), younger professionals (30, 42, 50, 52, 62), professionals with limited experience (30, 42), and those living with family members (31). Similarly, the risk of experiencing psychological stress or distress was higher among nurses (31, 42, 49, 50, 54, 65) and frontline professionals than among other HCWs (24, 31, 49). Prevalence of stress and distress was higher among frontline nurses (46%, 95% CI: 39–54%, *N* = 17) than among nurses working on the second line (42%, 95% CI: 31–53%, *N* = 20) (32). Similarly, frontline health professionals experience higher levels of distress (mean = 2.66 ± 0.93) than other health professionals (mean = 2.46 ± 0.83) (42). The disproportionate need for technological supplies in ICU settings, combined with the scarcity of these supplies, promotes high rates of psychological stress among HCWs who work in ICU settings (41). Similarly, a lack of adequate PPE (24), direct exposure to patients (54, 58, 62), working in ICU or emergency settings (42), working in a perceived unsafe environment (30), working in COVID-19 hospitals (62), and working in regions with high caseloads (49) were associated with an increased risk of developing stress or distress. Emotional stress was also associated with a lack of training and social support (30) and a history of mental illness or chronic disease (24, 42).

Varghese et al. (62) examined the pooled prevalence among nurses across various regions. The highest prevalence was reported for the Eastern Mediterranean region (61.6%, 95% CI: 56.4–66.8%, *N* = 2, *n* = 763) compared with the Western Pacific/Southeast region (47.2%, 95% CI: 14.7–81%, *N* = 4, *n* = 3,165) and the European region (34.2%, 95% CI: 21.2–48.6%, *N* = 3, *n* = 232) (62).

#### Insomnia

Insomnia was assessed by 16 reviews, which synthesised data from 91 primary studies (Table 2). The prevalence rate was reported in all 16 reviews, including 9 that reported pooled prevalence values calculated from meta-analyses, ranging from 23.98% (95% CI: 16–32%, *N* = 4) (52) to 47.3% (95% CI: 39–56%, *N* = 7) (65). The most-reported insomnia assessment tool was the ISI, which was reported in 7 reviews (Table 2).

Insomnia risk factors include female gender (24, 50), occupation as a nurse (50, 65), being a frontline professional (24, 42, 50), existing organic illness (24), and younger age (≤30 years) (52). Additionally, direct exposure to a COVID-19 patient (54), fear for self-infection (54, 58), working in an isolation unit (54), living in a rural area (24), and a lack of faith in psychological support (54) were associated with the increased incidence of insomnia.

#### Burnout

Burnout was assessed from 8 reviews, which synthesised data from 62 primary studies (Table 2). Of these, the prevalence rate was reported in 6 reviews, and only 1 study reported the pooled prevalence from a meta-analysis (43), which indicated an overall pooled prevalence for burnout of 25% (95% CI: 13–43%, *N* = 3) (43). Other reviews reported estimated prevalence values ranging from 12% (42) to 45.6% (25). The prevalence of burnout domains was reported in one review (44), which indicated that emotional exhaustion (34.1%), depersonalisation (12.6%), and lack of personal accomplishment (15.2%) were common reasons cited for burnout among nurses (*N* = 6). The most-reported burnout assessment tool was the Maslach Burnout Inventory (MBI), which was reported in 4 reviews (Table 2).

Burnout prevalence was higher among women (42, 50, 60) and younger professionals (44, 54). Decreased social support (44), fewer years of experience (<5 years) (60), more time spent working in quarantine areas (44), working in high-risk environments (44), working with insufficient resources (44), increased workload (44), and lower levels of specialised training (44) were significant risk factors for burnout. Among various health professionals, nurses (42, 54, 60) and frontline HCWs (42) were more at risk of developing burnout than other health professionals.

#### Other Mental Health Impacts

Other reported mental health impacts associated with the COVID-19 pandemic included fear of infection (4 reviews, *N* = 26), obsessive-compulsive disorder (2 reviews, *N* = 5), phobia (1 review, *N* = 4), somatisation symptoms (2 reviews, *N* = 6), substance abuse (1 review, *N* = 1), and suicidal ideations or self-harm (1 review, *N* = 4) (Table 2).

The fear of infection ranged from 60 to 96.6% (*N* = 12) among dental professionals (33). Additionally, a prevalence of 77.1% (*N* = 4, *n* = 3,558) for fear of infection was reported in Asia (61). One review (45) reported pooled prevalence values for obsessive-compulsive disorder (16.2%, 95% CI: 3–30%, *N* = 4), phobias (35%, 95% CI: 8.6–61, *N* = 4) and somatisation symptoms (10.7%, 95% CI: 1.9–19.6%, *N* = 5), and another review (52) reported a pooled prevalence for suicidal ideation (5.8%, 95% CI: 5–7%, *N* = 4). The prevalence of substance abuse was reported to be 6.2% among nurses and doctors in Spain (25).

#### Interventions/Coping Strategies Reported Alongside the COVID-19-Related Mental Health Issues

Strategies for overcoming mental health problems encountered during the COVID-19 pandemic included identifying people at risk (61), seeking individual or group-level professional psychological support (42, 51), attending counselling (51), practising mindfulness exercises (61), pursuing religious or spiritual channels (42), obtaining online information (51), refocusing and performing positive appraisal (42), ensuring family safety (24), seeking support from families or relatives (51, 61), asking for support from nurse leaders (60), practising resilience (24, 61), being in a committed relationship (24, 61), attending training or orientation for infectious disease unit (24, 60, 61), verifying access to adequate PPE (24, 51, 60, 61), reducing workloads (57), and reducing job-related stressors (57). One review reported participants, who prefer to overcome their psychological distress alone without any intervention (51).

## Discussion

To our knowledge, this is the first meta-review to investigate the impacts of COVID-19 pandemic on the overall mental health and well-being of HCWs (allied health professionals, doctors, and nurses). One strength of this meta-review is the large sample size included, which was drawn from 1,828 individual studies performed worldwide to evaluate the psychological impacts of COVID-19 on health professionals.

The most prevalent mental health problems identified in this review included anxiety, depression, and stress/PTSD. Other prevailing mental health problems include burnout, insomnia, fear of infection, obsessive-compulsive disorder, phobia, somatisation symptoms, substance abuse, and suicidal ideation/self-harm. Significant risk factors associated with the incidence of mental health issues include female gender, young age, low educational level, being a nurse, being a frontline health professional, experience, and country of residence. This meta-review reports the most comprehensive evidence to date regarding the mental health prevalence and risk factors among global HCWs associated with the COVID-19 pandemic. Mental health is among the commonly reported concerns associated with COVID-19 (4–6), particularly among individuals in the general population who have limited knowledge regarding the pandemic and tend to experience a high prevalence of adverse mental health conditions (4). Although the healthcare professions have stronger knowledge and experience in managing the pandemic condition, their mental health concerns are no different, or even higher than the general population. Accordingly, the overall pooled prevalence of mental health issues was reported to be higher among HCWs, compared to the general population (27, 39) but lower than that among COVID-19 patients (46). Additionally, hard-affected countries, such as Italy (25), were associated with a higher prevalence of mental health issues relative to other regions. During the early stages of the outbreak, the highest prevalence of mental health issues was reported in Hubei Province, China, where the outbreak originated (4). Similar to the COVID-19 outbreak, previous pandemics, including SARS and MERS, were also characterised by mental health disturbances among health professionals (10, 11).

The findings of this meta-review further indicated that female HCWs are at a greater risk of mental health concerns than their male counterparts, which was identified for anxiety, depression, stress, insomnia, and burnout. Although none of the studies included in this review examined the nature of this association, the additional domestic burden among women has reportedly increased during COVID-19, including childcare, which likely contributed to worse mental health (66). Bahrami et al. (67) were of the opinion that metacognitive belief in uncontrollability, advantages, and the avoidance of worry may have contributed to the higher prevalence of anxiety in women than in men. Similar patterns of increased psychological disturbances were observed among females in the general population (6, 68) and among other professionals, such as teachers (69) during the COVID-19 pandemic. Additionally, the study reported by Hou et al. (68) examining differences during the COVID-19 pandemic indicated that men showed more resilience to stress, whereas women experienced more stress and anxiety symptoms.

Anxiety was the most prevalent mental health problem reported among HCWs during the COVID-19 pandemic, according to the findings of this review. The highest reported anxiety prevalence was 65.2% (25). The prevalence of anxiety varies across professions, with nurses reporting higher levels of anxiety than other professionals, which might be attributable to nurses having more frequent contact with the patients relative to other health professionals. Various studies have reported severe or dysfunctional anxiety levels among nurses due to the nature of various nursing roles (24, 65, 70). A similar prevalence of anxiety has been reported among teachers during the COVID-19 pandemic (69). The review by de Oliveira Silva et al. (69) reported an anxiety prevalence between 10 and 49.4% among teachers, which was associated with workload and the demand for online teaching. Higher anxiety was also found among pregnant women during the third trimester of pregnancy, associated with poor social support and increased demand on them to use COVID-19 protective measures (71). The causes of increased anxiety are likely multifaceted and are further complicated by the impacts of the pandemic.

The findings of this meta-review further indicated that the highest prevalence of depression was reported at 65% (42). Unsurprisingly, the rate of depression was higher among professionals in contact with COVID-19-positive patients and those working in COVID-19 units (24, 42, 50, 63, 65), which is likely to be associated with increased interaction with dying or suffering patients. Additionally, professionals with insomnia were 13 times more likely to develop depressive symptoms than those without insomnia (39). Increased depression incidence may be associated with a fear of contracting the infection or infecting family members, as has been reported in some studies (33, 51). A recent review study examining frontline professionals also indicated an association between depressive symptoms and the direct diagnosis or treatment of COVID-19 patients (5). High rates of depression or depressive symptoms have also been reported among the general population (4, 6), which has been associated with increased alcohol use (4) and suicidal ideation (6).

Stress-related symptoms were identified as common psychological concerns among HCWs. The findings of this meta-review indicated various emotional stress conditions associated with COVID-19, including acute stress, distress, and PTSD symptoms. The prevalence was reported as high as 78% for distress and 71.5% for PTSD. Stress, including PTSD in particular, may be associated with the exposure of HCWs exposure to adverse conditions, coupled with the increased demand for work. Previous studies conducted during pandemics also reported that HCWs in emergency units were exposed to traumatic stressors, such as the burden of rapid decision-making, demands to manage patient and family expectations, unexpected daily caseloads, and high fatality rates (9, 72). The pattern of stress identified among HCWs in the current review is similar to that described by teachers (69). Similar to anxiety and depression, being a nurse or frontline professional was identified as a significant risk factor for stress associated with COVID-19. In line with previous studies, the burden of stress among HCWs may be influenced by poor social support, coupled with fear of getting infected or infecting family members (9, 70, 71).

The findings of the current review further indicate differences in the mental health concerns of health professionals across regions. For instance, in China, HCWs in various provinces were reported to experience less anxiety than those working in Hubei Province, where the outbreak originated (30.8 vs. 37.9%). The current review further identified that the three most commonly occurring psychological concerns (anxiety, depression, and stress) were experienced at higher rates in some countries than in others. The highest prevalence of anxiety was reported in the UK (22%), whereas the highest prevalence of depression was reported in the Middle East (41%), and the highest stress level was observed in the Eastern Mediterranean region (61.6%). By contrast, the lowest prevalence of anxiety was reported in Italy (13.44%), the lowest prevalence of depression was reported in the UK (19.29%), and the European region experienced the least stress (34.2%). Previous studies indicate that higher levels of mental health concerns observed in particular regions or countries may be associated with large caseloads or poorly functioning healthcare systems (4, 73).

Other mental health concerns identified in this meta-review include burnout, fear of infection, phobia, somatisation symptoms and substance abuse, each affecting more than one-quoter of the professionals except somatization symptoms. Of these, fear of infection is the most prevalent, with a prevalence rate of as high as 96.6% among dental professionals while somatization symptoms were the least reported mental health concern among the professionals, accounting for about 10%. Fear of covid-19 was reported to spread faster than the virus (13) and is strongly associated with the uncertainties about the outbreak, of which many countries, including high-income countries, are struggling to contain the outbreak (12, 13). On this note, Pakpoup and Griffiths (74) opined the need for understanding the different factors underpinning the fear associated with the virus to determine the needed education and prevention programs, and which groups of people to target. These programs could be instrumental towards overcoming the fear of COVID-19 and affected individuals to engage in preventative behaviours (74). Burnout on the other hand, may be associated with increased rates of hospitalisation coupled with longer working hours, particularly among frontline professionals. During the initial stages of the outbreak, burnout was highest among nurses, especially the depersonalisation sub-scale (75). This is largely associated with longer working hours, of which those with younger age were most affected compared to experienced and/or older professionals (75).

### Review Limitations

Although this meta-review provides comprehensive evidence regarding the overall mental health impacts of the COVID-19 pandemic among health professionals, various limitations must also be considered when interpreting these findings. First, many of the included systematic reviews were associated with the potential for bias, as assessed by the JBI systematic review checklist (36) (Table 1). However, this could be associated with the rapid nature of the pandemic evolution, coupled with the need to quickly fill research gaps. Second, systematic reviews both with and without meta-analyses were included in this meta-review; therefore, no additional meta-analyses were conducted. Instead, the findings were narratively synthesised, and the only effect sizes available are those that were reported by the included studies. Third, it is unclear from the included systematic reviews if the HCWs had underlying conditions prior to the COVID-19 pandemic, which may have exacerbated the development of the various mental health issues identified in this review. Finally, the current review only reported coping strategies identified alongside the prevalence and risk factors associated with the various mental health conditions. Additional studies remain necessary to specifically investigate interventional techniques capable of supporting the mental health of health professionals during pandemics such as COVID-19.

### Conclusions

Based on the findings of this meta-review, health professionals (nurses, doctors, and allied health professionals) experience various forms of COVID-19-related mental health issues. The most prevalent mental health issue is anxiety, followed by depression and stress/PTSD. Other significant mental health problems include insomnia, burnout, fear of infection, obsessive-compulsive disorder, somatisation symptoms, and suicidal ideation/self-harm. Female gender and younger age were the most significant sociodemographic risk factors associated with COVID-19-related mental health impacts. Other risk factors included being a nurse and being a frontline professional. The findings of this meta-review have implications for both practise and policies, therefore, we recommend targeted interventions and health programs that address specific mental health issues to support health professionals worldwide during pandemics such as COVID-19. This is in line with the position paper of the World Psychiatric Association (76), which recommended continued psychiatric support including telepsychiatry, promoting adherence to physical health measures such as social distancing, as well as respecting the human rights of individual with mental disorders. McDaid (77) added the need for strategies to support overall mental health recovery beyond the pandemic, which could be tailored to individual country context.

## Data Availability Statement

The original contributions presented in the study are included in the article/Supplementary Material, further inquiries can be directed to the corresponding authors.

## Author Contributions

MC, DS, and UB: conceptualisation and study protocol. PJ and MK: articles search. MC and PJ: article screening and selection. DS, LD, and PP: data extraction. NC, PJ, and TK: quality assessment. MC, AC, DS, and UB: data analysis. RM, KN, and PK: supervision and review for intellectual content. MC, DS, and DN: writing first draft of manuscript. All authors: final approval of manuscript.

## Funding

The work of AC and UB is supported by The Hong Kong Polytechnic University, Hong Kong SAR and The Government of the Hong Kong Special Administrative Region & Innovation and Technology Fund.

## Conflict of Interest

PJ was employed by Health Careers International Pty Ltd. The remaining authors declare that the research was conducted in the absence of any commercial or financial relationships that could be construed as a potential conflict of interest.

## Publisher's Note

All claims expressed in this article are solely those of the authors and do not necessarily represent those of their affiliated organizations, or those of the publisher, the editors and the reviewers. Any product that may be evaluated in this article, or claim that may be made by its manufacturer, is not guaranteed or endorsed by the publisher.
