# Supplementary material for: COVID-19 Pandemic and Overall Mental Health of Healthcare Professionals Globally: A Meta-Review of Systematic Reviews
Source: Front Psychiatry. 2022 Jan 17;12:804525. doi: 10.3389/fpsyt.2021.804525 (PMC8801501; doi:10.3389/fpsyt.2021.804525)
Supplement: Supplementary file 1 [file Table_1.DOCX]

| **Supplemental Table S1: Characteristics of Included Review** | | | | | | | |
| --- | --- | --- | --- | --- | --- | --- | --- |
| **No** | **Author** | **Year** | **Gender/age (years) or range OR mean age** | **Search strategy** | **Studies included/and participants** | **Outcomes/validated tool** | **Risk of bias/Instrument used** |
| 1 | Al Maqbali (32) | 2021 | Age: NR.  Sex: NR | **Databases used**: PubMed, CINAHL, Medline, Embase, PsycINFO, MedRxiv and Google Scholar.  **Search period**: January 2020 to October 2020.  **Eligibility**: 1) studies reporting the prevalence any of stress, anxiety, depression, or sleep disturbances on nurses. 2) All types of settings, 3) cohort or cross-sectional surveys. | Number of studies included: 93  Number of participants: 93,112  Population: Nurses  Design: Cross-sectional  Settings: Hospital (n=67); Mixed (n=17); undefined (n=9) | Depression, stress, anxiety, and sleep disturbances/validated tool (DASS-21, SCL-90, IES-R, GAD-7, PHQ-4, 9; PSS-4, ISI, SRQ-20, SAS, SDS, PCL-5, HADS, PSQI, PTSD, SOS, ASDI, BDI, HAM-A, STAI, SRSS, WSP, PDSS, BAI, CAS, AIS, and NSS). | Low-medium quality studies/NOS checklist |
| 2 | Arora (23) | 2020 | Age: 18-75  Sex: Male: (n=32,342, 33.3%), female (n=62,215, 62.3%): not reported: (n=4,319, 4.4%). | **Databases used**: PubMed, Medline, Web of Science, Embase, PsycINFO, Scopus, and CINAHL.  **Search period**: March-April, 2020.  **Eligibility**: 1) Human studies, 2) original research data, and 3) at least one psychological outcome reported. | Number of studies included: 30  Number of participants: 97,173 (General population n=81,664); healthcare professionals+general population (n=939); healthcare professionals (n=14,570).  Population: Health workers & general population  Design: Cross-sectional (n=29), collect data across two time points (n=1). | Depression, stress, anxiety, and PTSD//validated tools (n=26), self-developed questionnaires (n=4). | Good (n=14), fair (n=7), and poor (n=7)/SQAOC&C |
| 3 | De Brier (49) | 2020 | Age: NR  Sex: NR | **Databases used**: PsycINFO, Medline, and NIPH systematic and living map on COVID-19 evidence.  **Search period**: March-May 2020.  **Eligibility:** 1) active healthcare workers, 2) Mental health or psychological outcomes. | Number of studies included: 33  Number of participants: 29,266  Population: Healthcare workers  Design: 32 cross-sectional studies, and one uncontrolled before-and-after study. | Depression, anxiety, and acute stress/posttraumatic stress, mental health, psychiatric morbidity, Post-traumatic stress symptoms, sleep/Validated tools (PSS, IES, K-6; PEESMI, DASS-21; SF-36, GHQ-28, BDI, CVDTS, VAS, SRAS, GSES, PSQI, SRDS). | Very low-quality evidence/GRADE methodology |
| 4 | Cenat (27) | 2021 | Age: NR    Sex: NR | **Databases used:** Embase, Scopus, Medline, APA PsycInfo, CINAHL, and Web of Science.  **Search period:** May 12, 2020  **Eligibility: 1)** studies published either in the French or English language, 2) availability of empirical data on the prevalence of mental health symptoms/disorders. | Number of studies included: 55  Number of participants: 189,159  Population: General population (n=41,000), Health workers (n=148,159).  Design: Any study with empirical data | Depression, anxiety, insomnia, psychological distress, and PTSD/ Validated tools (BAI, **GAD-7**, DASS-21, BDI-II, WHO-5, SDS, PHQ-9, GAD-2, HADS, ISI, IES-R, K-6, PSQI, CPDI, GPS-PTSS, SCL-90, PGWB, PCL-C). | Good/ JBI-checklist |
| 5 | da Silva (42) | 2021 | Age: NR  Sex: NR | **Databases used:** LILACS, PsycINFO, Medline, and BMJ.  **Search period**: April-May 2020.  **Eligibility:** 1) healthcare professionals, 2) observational and cross-sectional studies, and 3) mental health variables assessed using validated instruments. | Number of studies included: 7  Number of participants: 7,102  Population: Healthcare professionals  Design: Cross-sectional & observational studies. | Depression, anxiety, stress, insomnia, psychological stress/ Validated tools (**GAD-7**, HAMA, HAMD, IES-R, ISI, PHQ-4, PHQ-9; SAS, SCL-90-R, SDS and PSQ). | NR |
| 6 | da Silva (39) | 2021a | Age: NR  Sex: NR | **Databases used:** Embase, PubMed, Scopus, and, ISI (Web of Science).  **Search period**: December 2019 to April 2020  **Eligibility:** 1) original studies on psychiatric repercussions on professionals (healthcare), 2) working to fight COVID-19, 3) observational studies. | Number of studies included: 8  Number of participants: health professionals (n=17,287), general audience (n=214)  Population: Health professionals & general audience  Design: observational studies. | Depression, anxiety, stress, insomnia, and distress/validated tools (PHQ-4, ISI, SAS, PSQI, VAS, GAD-7, AIS and SOS). | NR |
| 7 | da Silva (43) | 2021b | Age: NR  Sex: NR | **Databases used:** ScienceDirect, Scopus, Embase Pubmed, and Web of Science.  **Search period**: January 2020 to January 2021.  **Eligibility:** All studies on health workers working in ICU during the COVID-19 pandemic. | Number of studies included: 31  Number of participants: NR  Population: Health professionals in ICU.  Design: All designs | Depression, distress, and sleep problems, anxiety/not specified. | Moderate quality/Not specified. |
| 8 | Danet (44) | 2021 | Age: NR  Sex: NR | **Databases used:** PubMed, Scopus, and Web of Science.  **Search period:** 5^th^ and 6^th^ August 2020  **Eligibility:** 1) quantitative studies, 2) healthcare professionals, 3) articles published 2020 and beyond either in English, German, French, Portuguese, and Italian, 4) studies that met ethical requirements. | Number of studies included: 12  Number of participants: 6,236  Population: Healthcare professionals  Design: Cross-sectional studies | Depression, anxiety, Stress, sleep disturbance and burnout/validated tools (**GAD-7**, STAI, ISI, PSQI, MBI, PHQ, SF-36, and IPQ). | Optimal quality/ PRISMA and AMSTAR-2 items checklist for critical evaluation of the systematic review. |
| 9 | De Kock (24) | 2021 | Age: NR  Sex: NR | **Databases used:** Embase, PROSPERO, Medline, HMIC, PsychInfo, Social Care Online, CINAHL, APA PsychInfo, SCOPUS, Business Source Elite, the King’s Fund Library, Health Source and Academic Search Complete and Google  **Search period:** Advanced.April-May 2020.  **Eligibility:** 1) primary observational & experimental studies which are qualitative or quantitative, 2) healthcare professionals | Number of studies included: 24  Number of participants: 13,731  Population: Health & Social care workers.  Design: qualitative & quantitative | Symptoms of Obsessive-Compulsive Disorder (OCD), depression, insomnia, anxiety, or distress/validated tools (PHQ-9, DASS-21, IES-R, **GAD-7**, VTQ, GSES, Kessler K6 Distress Scale, PSQI, CES-D, SDS, SOS, and DAS). | Fair quality for cross-sectional studies, low risks of bias for the majority of studies, qualitative studies has good qualities/JBI-checklist, Critical Appraisal Skills  Programme (CASP) appraisal tool, ROBINS, and MMAT. |
| 10 | de Pablo (57) | 2021 | Age: Mean age was 36.1±7.1 years; ranges from 23-69.4years.  Sex:females (n=49,697, 77.1%); males (n=14,761, 22.9%) | **Databases used:** The Russian Science Citation Index, the BIOSIS Citation Index, Web of Science Core Collection, the KCI-Korean Journal Database, MEDLINE, and the SciELO Citation Index,  **Search period:** from inception. until 15th April 2020.  **Eligibility:** 1) Primary studies, 2) healthcare professionals exposed to COVID-19, ARS/MERS, 3) mental or physical health outcomes, 4) written in the English language, and 5) have >5 samples. | Number of studies included: 115  Number of participants: 64,458.  Population: healthcare professionals.  Design: quantitative & qualitative study designs. | Psychological distress, anxiety, depressive symptoms, PTSD, burnout, fear, stigmatisation feelings, general health concerns, Insomnia, and somatisation/ Self-administered questionnaires, interviews/evaluations, or surveys. | Quality ranged from 1-5 (MMAT score) |
| 11 | d’Ettorre (30) | 2021 | Age: NR  Sex: NR | **Databases used:** MEDLINE/Pubmed  **Search period:** February-October  2020  **Eligibility:** Post-traumatic stress in healthcare workers | Number of studies included: 17  Number of participants: 32,196  Population:: healthcare professionals.  Design: cross-sectional studies | Post-traumatic stress/PTSD-cheklist, CBI, IES-R | Moderate quality/NOS |
| 12 | Dong (34) | 2021 | Age: NR  Sex: NR | **Databases used:** Data, Chongqing VIP, PubMed, PsycINFO, Embase, Wanfang, Sinomed, and Chinese National Knowledge Infrastructure databases.  **Search period:** January-October 2020  **Eligibility:** Chinese health workers irrespective of age, being of any ethnicity, and studies that are cross-sectional, observational or longitudinal, published papers with outcomes of stress, depression and anxiety. | Number of studies included: 25  Number of participants: 30,841  Population: healthcare workers  Design: cross-sectional studies | Depression, anxiety, and stress/validated tools (SAS, SCL-90, SDS, PHQ-9, **GAD-7**, PQEPHE, SQR-20, Fear NRS, PTSD-SS) | High quality (n=23, 88%), moderate quality (n=3, 12.0%)/AHRQ 11-item checklist |
| 13 | Falasi (31) | 2021 | Age:19-50years  Sex: NR | **Databases used:** Medline, Health Psychosocial Instrument (HaPI), PsychINFO, and Embase.  **Search period:** January 2003-November 2020  **Eligibility:** 1) studies on healthcare professionals, published from 2003-2020, in the English language. | Number of studies included: 8  Number of participants: 10,074  Population: healthcare workers  Design: cross-sectional studies | Post-traumatic stress/validated tools (PCL-6, IES-R, IES-15). | Good/JBI-checklist |
| 14 | Galanis (58) | 2021 | Age: 31-42.4years  Sex: NR | **Databases used:** Scopus, PubMed, ProQuest, CINAHL, and Cochrane COVID-19 registry.  **Search period:** January-November 2020.  **Eligibility:** studies published in the English language, assessing burnout and its associated factors in the COVID-19 era using validated outcome measures, on nurses working in hospitals. | Number of studies included:16  Number of participants: 18,935  Population: healthcare workers  Design: cross-sectional studies | Burnout/validated tools (OLBI, Mini-Z, and SBI). | Not clearly defined/JBI-checklist |
| 15 | Gohil (33) | 2021 | Age: NR  Sex: NR | **Databases used**: Medline/Pubmed, Proquest databases, and Embase.  **Search period**: January 2020 to April 2021.  **Eligibility**: 1) studies published in the English language, 2) Assessing the impact of COVID-19 on the psychological health of dental practitioners. | Number of studies included: 20  Number of participants: 18,222  Population: healthcare workers  Design: cross-sectional studies | Depression, anxiety, fear, and uncertainty/Unspecified self-reported measures | Not reported |
| 16 | Hao (45) | 2021 | Age: NR  Sex: female (n=7,620, 70%); male (n=3,266, 30%). | **Databases used**: Chinese databases (Chinese Biomedical Literature Database,  China Science and Technology Journal Database, China National Knowledge Infrastructure and Wanfang database), PubMed, Scopus, EMBASE, and PsycINFO.  **Search period**: January-April 2020  **Eligibility**: 1) healthcare professionals, 2) involved in the care of people suspected or confirmed to have had COVID-19, 3) reported the prevalence of mental health problems, 4) using cross-sectional or cohort study designs and 5) published in a peer-reviewed journal. | Number of studies included: 20  Number of participants: 10,886  Population: healthcare workers  Design: cross-sectional | Depression, phobia, insomnia, anxiety, post-traumatic stress symptoms, obsessive-compulsive symptoms, and somatization symptoms/validated tools (SCL-90, PHQ-9, PSQI, SAS, PTSD-SS, **GAD-7**, ISI, SQR-20, IES-R, PCL-C, HAMD, HAMA, DASS-21, SDS, and PSS-10). | Moderate quality/AHRQ 11-item checklist |
| 17 | Krishnamoorthy (63) | 2020 | Age: mean age 30.6 to 49.9 years  Sex: NR | **Databases used**: Chinese, Google Scholar, Medline, national knowledge  infrastructure, Cochrane library, and ScienceDirect.  **Search period**: until 22 April 2020.  **Eligibility**: 1) all studies irrespective of settings, 2) studies on healthcare professionals and the general population, 3) assessing psychological outcomes and impact of events. | Number of studies included: 50  Number of participants: 171,571  Population: general population & healthcare professionals.  Design:cross-sectional studies. | sleep quality, stress, psychological distress, insomnia, post-traumatic stress symptoms, anxiety, depression/ validated tool (PCL-C, **GAD**, PHQ, SDS  SAS, GHQ, PSS, WHO, CES-D, PSQI, PTSD-SS  HADS, IES-R, ISI, SRQ, HAMD, HAMA, SOS, AIS, CPDI, GPS, DASS, GSI). | High risks of bias/NO scale |
| 18 | Kunz (25) | 2021 | Age: NR  Sex: Female (n=33,450). | **Databases used**: NR    **Search period**: March 2020 to January 2021.  **Eligibility**: 1) original studies conducted in Europe, North America and Australia, 2) conducted among healthcare professionals, 3) using a validated outcome measure. | Number of studies included: 27  Number of participants: NR  Population: nurses & doctors.  Design: unspecified | Depression, anxiety, stress, sleep, post-traumatic stress, burnout, substance use disorder, and somatization/validated tools (HADS, PHQ-9, PHQ-8, DASS-21, BDI, PHQ-2, **GAD-7**, GAD-2, SAS, HARS, ISI, IES-R, PSS-14, STAI-Y, EASE, GPS, PCL-5, PDEG, ProQOL-5, MBI, PFI, CAGE-AID). | NR/JBI-checklist |
| 19 | Kunzler (64) | 2021 | Age: 20 to 56.9  Sex: Female (n=9,648, 4.6%). | **Databases used**: PsycINFO, PubMed, and Web of Science.  **Search period**: January 2019 to May 2020,  **Eligibility**: 1) studies on the general population, healthcare professionals or patients categories irrespective of their age, employment or health status, 2) original research articles (cross-sectional, and longitudinal surveys). | Number of studies included: 104 (n=50, general population n=30, other patient groups n=7).  Number of participants: 208,261  Population: general population and healthcare professionals.  Design: cross-sectional, observational, and controlled studies. | Anxiety, worries, fear, depression, stress, insomnia, post-traumatic stress, and psychological distress/validated tools (AIS, BDI, BRCS, CD-RISC, CES-D, PQ-5, ScoVGAD-7, DASS-21, EPDS, EPDS-3A, FACIT-Sp12, FCV-19S, GAD, GPS-PTSD, GSES, ISI, HADS, HAI, HAMA, HAMD, IES-R, IUS-12, JGLS, K-6, MINI, MSPSS, PANSS, PCL-5(−C), PHQ-2, PROMIS-SFs, PSQI, PSS, PTSD-SS, Ryff’s PWB Ryff’s, SASR, SCL-90, SCS, SDQ, SDS, SF-12(/−36), SHAI, SOS, SPEQ, SRQ, STAI-Y, SSRS, SSS, SWLS, TAF, TEMPS-A, PISA, VDAS, VAS, WHO-5, WHOQOL-BREF, 4−/5−/7−/11-P LS, and 4−/5−/6−/11-point Likert-scale. | Fair quality=38, poor quality=57, fair quality=7 /Modified National Institutes of Health (NIH) Quality  Assessment Tool for Observational Cohort and Cross-Sectional Studies |
| 20 | Luo (56) | 2021 | Age: NR  Sex: NR | **Databases used**: PubMed, WHO COVID-19 databases, Embase, and Google scholar.  **Search period**: November 2019-May 2020.  **Eligibility**: primary studies assessing psychological distress on the general population, patients with pre-existing conditions, and healthcare professionals. | Number of studies included: 62  Number of participants: 162,639  Population: healthcare professionals and the general population.  Design: cross-sectional or cohort studies | Depression, and anxiety, and distress.Validated tools (BDI, DASS-21, HAMD, HADS, PHQ4/9, and SDS) | Moderate to high quality/11 studies scored 10 out of 12, 22 studies scored 11, and 29 studies scored 12 (/McMaster University critical appraisal tool |
| 21 | Mahmud (28) | 2021 | Age: 15-28years  Sex: males (n=43,351, 30%); females (n=101,118, 70%). | **Databases used**: MEDLINE, PubMed, Web of Science, and Google Scholar databases.  **Search period**: March 30, 2021.  **Eligibility**: 1) studies on the prevalence of depression, anxiety, and insomnia, 2) health workers, 3) in the English language, 4) observational studies, and 5) with full-text available. | Number of studies included: 69  Number of participants: 144,649  Population: healthcare professionals  Design: cross-sectional study | Depression, anxiety, insomnia/ validated tools (CES-D, DASS-21, HADS, PHQ, and SDS, ISR, HAMD, FCV-19S, ISR, PDI, PHQ-4, PSSQ EASE, PHQ-15, SAVE-9, BAI, ISI, PSQI, **GAD**, HAMA, IES, PSS, PTSD-8, and AIS. | Average STROBE score 21.54/STROBE checklist |
| 22 | Marvaldi (26) | 2021 | Age: NR  Sex: Female (n=71,051, 70.3%); male (n=29,966, 29.7%). | **Databases used**: Pubmed and PsycINFO.    **Search period**: up to October 2020.  **Eligibility**: 1) studies evaluating the effects of mental health on healthcare personnel, 2) using validated outcome measures, 3) published until October 2020. | Number of studies included: 70  Number of participants: 101,017  Population: healthcare workers  Design: cross-sectional studies | Anxiety, depression, ASD, and PTSD, insomnia, burnout, acute stress syndrome, and distress /validated tools (SAS, SDS, PSQI, PSS, **GAD** (2, 7), PHQ (2,9), HAMA, HAMD, ISI, HADS, CES-D, BDI-II, SCL-90-R, DASS-21, IES-R, GHQ-12, ProQol/burnout, PC-PTSD, CPSS, PCL-5, Mini-Z burnout, EE, DP, MBI, SASRQ, and Psychosomatic - OCS – Phobic. | High quality/AHRQ 11-items checklist. |
| 23 | Moitra (46) | 2021 | Age: NR  Sex: Female (n= 64%) | **Databases used**: PubMed and Embase.  **Search period**: Dec 2019–June 2020.  **Eligibility**: 1) studies reporting qualitative and quantitative data reporting mental or psychological healthcare on healthcare professionals. | Number of studies included: 51  Number of participants: overall samples not reported; individual studies samples ranged from 52 to 14,825.  Population: healthcare professionals.  Design: cross-sectional studies (88%). | Anxiety symptoms, depressive symptoms, sleep quality, psychological trauma, insomnia, workplace burnout, fatigue, and distress/validated tools (PHQ, and **GAD** were the most commonly used measures; others were the PSQI). | NR |
| 24 | Muller (60) | 2020 | Age: NR  Sex: NR | **Databases used**: PubMed, Embase, and Centers for Disease Control and Prevention.  **Search period**: up to May 2020.  **Eligibility**: 1) All types of studies involving healthcare workers, 2) mental health outcomes. | Number of studies included: 59  Number of participants: 54,707  Population: healthcare workers  Design: cross-sectional studies (n=46, 78%) | Sleep problems, depression, anxiety, and distress/NR | Low methodological quality (n=25), medium (n=12), and high quality (n=16)/Quality of systematic reviews (AMSTAR tool), qualitative studies (CASP checklist), cross-sectional studies (JBI checklist), and  the overall evidence is very low (GRADE methodology) |
| 25 | Pappa (29) | 2020 | Age: NR  Sex: NR | **Databases used**: PubMed, MEDLINE, and Google Scholar databases  **Search period**: up to April 2020  **Eligibility**: 1) Studies evaluating the prevalence of depression, anxiety and insomnia using a validated outcome measures | Number of studies included: 13  Number of participants: 33,062  Population: healthcare professionals  Design: cross-sectional studies | Depression, anxiety, and insomnia/validated tool (DASS-21, **GAD** (2, 7), PHQ (2,9), BDI, SAS, SDS, CES-D, BAI, ISI, PSQI, AIS, HAMA, HAMD). | Fair to good quality/Modified Newcastle-Ottawa quality assessment scale |
| 26 | Phiri (48) | 2021 | Age: NR  Sex: NR | **Databases used**: PubMed, Proquest, EBSCOhost, and ScienceDirect.  **Search period**: April 2020 to January 2021.  **Eligibility**: 1) qualitative, quantitative and empirical studies on the mental health of the healthcare professionals, general public and the patient's population. | Number of studies included: 287  Number of participants: 1,023,010.  Population: healthcare professionals and the general public.  Design: cross-sectional, longitudinal, case, retrospective, pilot, longitudinal, case-control, cohort, comparative, and review studies | Depression, anxiety and PTSD, suicidal ideation/thought, and self-harm/validated tools (**GAD** (2, 7), PHQ (2, 4, 8, 9), ISI, HAMA, HAMD, SDS, SAS, PSQI, PCL-C, PCL-5, DASS-21, IES-R, SCL-90-R, DSM-5 diagnostic criteria for BPD, CES-D, HADS, WHO-5, BAI, BDI, SHAI, CoVGAD-7, AUDIT (C, 5), WEMWBS, SHAI, PGWB, CoVGAD-7, Quantity/Frequency/Peak Alcohol Use Index, TLFB, STAI, Gallup-Healthways Well-Being Index, SDS, YSIS, IPAQ-SF, BSI-18, GADS, SCI, IPAF-SF, PCL-6, DASS-9, MOSS-SS, DASS-21, IES-R, K10, GSI, OCD, ESS, SQS, MFQ, MHI-5, QIDS-SR16, HSCL, OCD, PMIR, M.I.N.I, SPRINT, ISO-30, OCIR, PTSD checklist, HARS, MADRS, GHQ-12, PC-PTSD, PANSS, YMRS, Negative and positive affect scales, TAF, GHQ-28. | NR/NOS |
| 27 | Salari (52) | 2020 | Age: 25 to 70 years  Sex: males (n=4,830, 21.6%), females (n=14,474, 64.7%), not reported (n=3,076, 13.7%). | **Databases used**: MagIran, SID, IranDoc, IranMedex, ScienceDirect, Scopus, Embase, Web of Science (ISI) PubMed, and Google Scholar  **Search period**: December 2019 to June 2020.  **Eligibility**: 1) studies assessing the prevalence of anxiety, depression, and stress among healthcare personnel. | Number of studies included: 29  Number of participants: 22,380  Population: healthcare professionals  Design: NR | Depression, anxiety, and stress/validated tools (SDS,  SAS, SASR, DASS-21, BDI-II, BAI, PSS, HAD, **GAD-7**). | High quality/STROBE checklist |
| 28 | Sanghera (50) | 2020 | Age: NR  Sex: NR | **Databases used**: Ovid Medline and Embase databases.  **Search period**: December 2019 to June 2020.  **Eligibility**: 1) studies evaluating the impact of SARS-CoV-2, 2) use of validated or self-developed outcome measures, 3) in the English language, 4) in the hospitals. | Number of studies included: 44  Number of participants: 69,499  Population: healthcare professionals  Design: cross-sectional studies. | Depression, anxiety, stress, burnout, insomnia, and post-traumatic stress/validated tools (most commonly used tools were **GAD-7**, ISI, IES-R, PHQ-9, EE, DP, PA, and DASS-21). | NR |
| 29 | Santabarbara (53) | 2021 | Age: 29 to 47 years  Sex: Females (n=39,643, 67.7%), males 18,922, 32.3%) | **Databases used**: Medline and PubMed.  **Search period**: December 2019 to September 2020  **Eligibility**: 1) cross-sectional studies reporting anxiety associated with COVID-19, 2) healthcare professionals, and 3) the assessment methods was described. | Number of studies included: 71  Number of participants: 58,565  Population: healthcare professionals  Design: cross-sectional studies | Anxiety/validated tools (HADS, SAS, **GAD-7**, HAMA, and DASS-21). | Ranged from 5-9/JBI checklist. |
| 30 | Saragih (65) | 2021 | Age: 18 to 50years  Sex: Female (n=40,338, 75%), males (n=13,446, 25%). | **Databases used**: MEDLINE, PubMed, Cumulative Index to Nursing and Allied Health Literature (CINAHL), Academic Search Complete, Web of Science, and SocINDEX.  **Search period**: up to November 2020.  **Eligibility**:1) healthcare professionals, 2) published in the English language, 3) the design should be case-control, cross-sectional or case-control studies. | Number of studies included: 38  Number of participants: 53,784  Population: healthcare professionals  Design: cross-sectional and case studies. | Depression, anxiety, distress, and post-traumatic stress/validated tools (NR). | High/JBI checklist |
| 31 | Li (51) | 2021 | Age: mean age ranges from 29 to 52.3  Sex: Female (n=60,525, 62.2%), males (n=36,808, 37.8%). | **Databases used**: MEDLINE, EMBASE, PsycINFO, Web of Science, Global Health, Google Scholar, CINAHL, and the Chinese databases SinoMed, WanfangMed, CNKI and CQVIP.  **Search period**: December 2019 and August 2020.  **Eligibility**: articles published in Chinese or English languages, 2) conducted on healthcare professionals, 3) reporting the prevalence of depression, anxiety and PTSD, 4) using diagnostic interviews or self-reported measures, | Number of studies included: 55  Number of participants: 97,333  Population: healthcare professionals  Design: cross-sectional | Depression, anxiety and PTSD/validated tools (PHQ (2, 4, 9), HADS, DASS-21, SDS, CES-D). | Low to high-quality studies/risk of bias tool. |
| 32 | Sharifi (61) | 2021 | Age: NR  Sex: NR | **Databases used**: MEDLINE via PubMed), Scopus, and Science Direct.  **Search period**: December 2019 to August 2020.  **Eligibility**: 1) articles published in the English language, 2) assessing burnout among healthcare professionals, 3) on healthcare professionals. | Number of studies included: 12  Number of participants: NR  Population: healthcare professionals  Design: cross-sectional studies | Burnout/validated tool (MBI). | Low-quality evidence/American Academy of Neurology criteria for the classification of evidence in causation studies. |
| 33 | Shaukat (40) | 2020 | Age: NR  Sex: NR | **Databases used**: PubMed and Google Scholar  **Search period**: January to March 2020  **Eligibility**: 1) articles published in the English language, 2) healthcare professionals. | Number of studies included: 10  Number of participants: 5,410  Population: healthcare professionals  Design: cross-sectional studies | Depression, anxiety, insomnia, and distress/validated tools (IES-R, PTSD-rating scale, ISI, **GAD-7**, SASR, Self-rating scale, GSES, and social support rating scale. | NR |
| 34 | Sheraton (62) | 2020 | Age: NR  Sex: NR | **Databases used**: Ovid, PubMed, Google Scholar and Psych.  **Search period**: November 2019 to May 2020.  **Eligibility**: 1) observational studies published in the English language, and 2) healthcare professionals. | Number of studies included: 14.  Number of participants: ranges from 174 to 4986.  Population: healthcare professionals.  Design: cross-sectional studies. | Stress, anxiety, depression, PTSD, social support, self-efficacy, and insomnia/validated tools (SAS, SSRS, GSES, SASR, PSQI). | High risks of bias/Cochrane risk of bias tool |
| 35 | Sriharan (59) | 2021 | Age: NR  Sex: NR | **Databases used**: Embase, Medline, CINAHL, PsycINFO, and ERIC.  **Search period**: until June 12, 2020.  **Eligibility**: 1) quantitative studies published in the English language, 2) studies on nurses. | Number of studies included: 9  Number of participants: NR  Population: nurses  Design: cross-sectional studies. | Burnout and moral distress/validated tools (MBI, Oldenburg Burnout Inventory, EE). | NR |
| 36 | Thatrimontrichai (41) | 2021 | Age: NR  Sex: NR | **Databases used**: PubMed.  **Search period**: December 2019 to September 2020.  **Eligibility**: 1) full published articles, irrespective of design or publication status. | Number of studies included: 32  Number of participants: 51,748  Population: healthcare professionals.  Design: unspecified | Depression, anxiety, fear, stress, insomnia, suicidal thought/ideation, somatic symptoms, psychological distress, Post-traumatic stress symptoms, obsessive-compulsive/validated tools (NR). | NR |
| 37 | Varghese (47) | 2021 | Age: 21-45 years  Sex: Females (n=12,522, 91.8%), males (n=1,119 ,8.2%). | **Databases used:** PUBMED, MEDLINE, Psych Info, Google Scholar, Nursing and Allied Health Database, Science Direct, Corona Virus Research Data­base and Web of Science Core Collection.  **Search period**: March to October 2020.  **Eligibility**: 1) nurses working in a hospital anywhere in the world, 2) exposure or interventions using a validated outcome measure for the assessment of mental health, 3) published in the English language. | Number of studies included: 25  Number of participants:  13,641  Population: nurses  Design: cross-sectional studies. | Depression, anx­iety, stress, PTSD, and insomnia/validated tools (**GAD**, SAS, CAS, GSI, STAI, HADS, SASRQ, GSI, PHQ-9, DASS-21, IES, PCL-C, PSS, HAMD and Self-reported Stressor and In­cidence Questionnaire). | Moderate to high quality/Loney criteria |
| 38 | Vindegaard (55) | 2020 | Age: 28.0-50.2  Sex: NR | **Databases used**: PubMed  **Search period**: unlimited  **Eligibility**: 1) studies reporting psychiatric symptoms or morbidity in patients with current or prior SARS-CoV-2 infection, or COVID-19, 2) infected and uninfected participants, 3) healthcare and non-healthcare professionals. | Number of studies included: 43  Number of participants: 43,691  Population: healthcare professionals, patients and the general public.  Design: a case-control study, and longitudinal survey. | Depression, anxiety, stress, post-traumatic stress, somatization, hostility, distress, phobia, paranoid ideation and psychoticism, specific phobias, cognitive changes, physical symptoms and loss of social functioning, sleep quality, social capital, dream anxiety, psychological wellbeing,/validated tools (WHO-5, DASS-21, ISI, IES-R, HADS, VDAS, SF-36, SRQ-20, **GAD-7**, PHQ-9, GHQ-12, Perceived severity, BSCS, PSCI-16, SAS, SASR, PSQI, K10, TEMPS-A, ASQ, PID-5-BF, HADS, HAI, CPDI, SF-12, K6). | NR |
| 39 | Wu (11) | 2021 | Age: NR  Sex: NR | **Databases used**: PubMed, Ovid, CNKI, Web of Science, and Wanfang Data) and preprint databases (SSRN, bioRxiv, Embase, and MedRxiv).  **Search period**: January to March 2020.  **Eligibility**: 1) Studies that are case-control, case-control, and cross-sectional studies, 2) using a validated outcome measure for assessing mental or psychological wellbeing, 3) including point prevalence data  for each outcome. | Number of studies included: 66  Number of participants: 221,970  Population: healthcare workers, and the general population.  Design: cross-sectional studies (95.5%). | Depression, anxiety, insomnia, and distress/validated tools (SDS, GHQ-9, 12, WHO-5, PSS-10, and K6). | Low risk of bias/STROBE checklist. |
| 40 | Zhao (38) | 2021 | Age: mean age ranges from 19.81 to 59.77  Sex: Females (n=42,153, 60.3%), males (n=26,873, 38.4%), not reported (n=913, 1.3%). | **Databases used:** EMBASE, PubMed, PsycINFO, Web of Science) and Chinese (China National Knowledge Internet [CNKI] and Wanfang databases).  **Search period**: up till May 2020.  **Eligibility**: 10 studies evaluating mental health comorbidities during COVID-19 or SARS, 2) availability of relevant data, 3) cross-sectional, case-control or cohort studies. | Number of studies included: 74  Number of participants: 69,939  Population: healthcare professionals and the general population.  Design: cross-sectional, longitudinal, and case-control studies. | Depressive symptoms, anxiety symptoms, stress, distress, insomnia, and post-traumatic stress symptoms, and poor mental health/validated tools (NR). | Moderate to high quality/Loney criteria |

^NOS=Newcastle-Ottawa Scale; SQAOC&C= Study Quality Assessment Tool for Observational Cohort & Cross-sectional Studies; PTSD=Post-traumatic stress disorder; PSS= Perceived Stress Scale; IES=Impact of Event Scale; K-6= Kessler Psychological Distress Scale; PEESMI= Professional burnout, Emotional Exhaustion Scale of the Maslach Burnout Inventory; DASS-21=Depression Anxiety Stress Scale; GHQ-12=12-item General Health Questionnaire; SF-36=Mental Component Summary of Short Form-36 instrument; GHQ-28=28-item General Health Questionnaire; BDI= Beck Depression Inventory; CVDTS=Chinese Version of Davidson Trauma Scale; VAS=Visual Analogue Scale; SRAS=Self Rating Anxiety Scale; GSES=General Self-Efficacy Scale; SASRQ=Stanford Acute Stress Reaction Questionnaire; PSQI=Pittsburgh Sleep Quality Index; VDAS=Van Dream Anxiety Scale; WHO-5=World Health Organization- Five Well-Being Index; WHOQOL-BREF=abbreviated World Health Organization Quality of Life; ISI=Insomnia Severity Index; CPSS=Chinese Perceived Stress Scale; SRAS=Self Rating Anxiety Scale; SRDS=Self Rating Depression Scale; NR=Not Reported; BAI=Beck’s Anxiety Inventory; EE=emotional exhaustion; DP=depersonalization; GAD-7=General Anxiety Disorder 7-items; WHO-5=WHO-Five Well- Being Index; PC-PTSD=4-item Primary Care PTSD screen; SDS= Self-Rating Depression Scale; GAD-2=General Anxiety Disorder 2-item; HADS=Hospital Anxiety and Depression Scale; IES-R=Impact of Event Scale-Revised; CPID=COVID-19 Pertitraumatic Distress Index; GPS-PTSS=Global Psychotrauma Screen, post-traumatic stress symptoms subscale; SCL-90=Symptom Check-List 90; SCL-90-R = Symptom Check List-90-revised; PGWB=Psychological General Well-Being questionnaire; PCL-C=PTSD Checklist– Civilian Version; HAMA= Hamilton Anxiety Scale; HAMD =Hamilton Depression Scale; AHRQ=Agency for Healthcare Research and Quality; PHQ= Patient Health Questionnaire; SAS = Zung's self-rating anxiety scale; PSQ=Psychological Stress Questionnaire; AIS=Athen’s Insomnia Scale; SOS=Stress Overload Scale; STAI=State-Trait AnxietyInventory; MBI=Maslach Burnout Inventory; IPQ=Illness Perception Questionnaire; VTQ=Vicarious Trauma Questionnaire; CES-D=Centre for Epidemiologic Studies Depression Scale; DAS=Dream Anxiety Score; CBI=Copenhagen Burnout Inventory; SRQ=Stress Reaction Questionnaire; PQEPHE=the psychosocial questionnaire of emergency public health events; PTSD-SS=post-traumatic stress disorder self-rating scale; PCL-6=Post-traumatic Checklist; OLBI=Oldenburg Burnout Inventory, SBI=Spanish Burnout Inventory; CASP=Critical Appraisal Skills Programme appraisal tool; JBI-checklist=Joanna Briggs Institute checklist; PSS-10=[Perceived Stress Scale; PSS-14= Perceived Stress Scale; NO= Newcastle-Ottawa scale; GSI=Global Severity Index; HARS=Hamilton Anxiety Rating Scale; STAI-Y= State Anxiety Inventory-trait form; EASE=Acute Stress of Health Professionals Caring COVID-19 scale; PCL-5= Post-traumatic Checklist-5; PDEG=Peritraumatic Dissociation Questionnaire; ProQOL-5=Professional Quality of Life Scale; PFI=Stanford Professional Fulfilment Index; CAGE-AID=Substance Abuse screening tool; BRCS=Brief Resilience Coping Scale; CD-RISC=Connor-Davidson Resilience Scale; PQ-5= Perception Questionnaire-5; SCoVGAD-7=Generalized Anxiety Disorder Scale-7 for COVID-19 Anxiety; CPDI CoViD-19=Peritraumatic Distress Index; EPDS=Edinburgh Postnatal Depression Scale; EPDS-3A=Edinburgh Postnatal Depression Scale-Anxiety subscale; FACIT-Sp12=Functional Assessment of Chronic Illness Therapy-Spiritual Well-Being Scale; FCV-19S=Fear of COVID-19 scale; GPS-PTSD=Global Psychotrauma Scale-posttraumatic stress disorder subscale; HAI=Health Anxiety Inventory; IUS-12=Intolerance of Uncertainty Scale-Short Form, JGLS=De Jong Gierveld Loneliness Scale; K-6(/− 10)=Kessler Psychological Distress Scale-6(/− 10); MINI=Mini International Neuropsychiatric Interview; MSPSS=Multidimensional Scale of Perceived Social Support; PANSS=Positive and Negative Syndrome Scale; PROMIS-SFs=Patient Reported Outcomes Measurement Information System short forms; Ryff’s PWB Ryff’s=Psychological Wellbeing Scales; SASR=Stanford Acute Stress Reaction; SCS=Self-Compassion Scale; SDQ=Strengths and Difficulties Questionnaire; SHAI=Short Health Anxiety Inventory; SPEQ=Specific Psychotic Experience Questionnaire; SSRS=Social Support Rating Scale; SSS=Somatic Symptom Scale; SWLS=Satisfaction With Life Scale; TAF=Triage Assessment Form; TEMPS-A=Temperament Evaluation of Memphis, PISA=Paris and San Diego-Anxious; STROBE checklist= Strengthening the Reporting of Observational Studies in Epidemiology (STROBE) statements; BSCS=Brief Self-Control Scale; K10=Kessler 10 Psychological Distress Scale; PID-5-BF=Personality Invemntory DSM-5-Brief-Form-Adult; ASDI=Acute Stress Disorder Inventory; SRSS= Sleep Self-Assessment Scale; WSP=Work Stress Profile; PDSS=Panic Disorder Severity Scale; CAS=Coronavirus Anxiety Scale.](https://www.corc.uk.net/outcome-experience-measures/perceived-stress-scale-pss-10/)^
